# Intraciliary calcium oscillations initiate vertebrate left-right asymmetry

**DOI:** 10.1016/j.cub.2014.12.051

**Published:** 2015-02-05

**Authors:** Shiaulou Yuan, Lu Zhao, Martina Brueckner, Zhaoxia Sun

**Affiliations:** 1 Department of Pediatrics, Yale University School of Medicine, 333 Cedar Street, New Haven, CT 06520, USA; 2 Department of Genetics, Yale University School of Medicine, 333 Cedar Street, New Haven, CT 06520, USA

## Abstract

**Background:**

Bilateral symmetry during vertebrate development is broken at the left-right organizer (LRO) by ciliary motility and the resultant directional flow of extracellular fluid. However, how ciliary motility is perceived and transduced into asymmetrical intracellular signaling at the LRO remains controversial. Previous work has indicated that sensory cilia and polycystin-2 (Pkd2), a cation channel, are required for sensing ciliary motility, yet their function and the molecular mechanism linking both to left-right signaling cascades is unknown.

**Results:**

Here, we report novel intraciliary calcium oscillations (ICOs) at the LRO that connect ciliary sensation of ciliary motility to downstream left-right signaling. Utilizing cilia-targeted genetically-encoded calcium indicators in live zebrafish embryos, we show that ICOs depend on Pkd2 and are left-biased at the LRO in response to ciliary motility. Asymmetric ICOs occur with onset of LRO ciliary motility, thus representing the earliest known LR asymmetric molecular signal. Suppression of ICOs using a cilia-targeted calcium sink demonstrates that they are essential for LR development.

**Conclusions:**

These findings demonstrate that intraciliary calcium initiates LR development and identify cilia as a functional ion signaling compartment connecting ciliary motility and flow to molecular LR signaling.

## Graphical Abstract

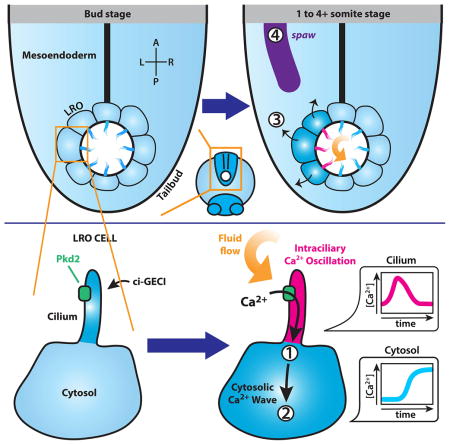

## Introduction

In vertebrates, cilia and polycystins are essential for the development of left-right (LR) asymmetry. LR asymmetry is initiated during the early somite stage at a conserved ciliated organ of asymmetry, the left-right organizer (LRO; also referred to as the ‘embryonic node’ in mouse, ‘Kupffer’s vesicle’ in zebrafish and ‘gastrocoel roofplate’ in frogs). Here, the inherent chirality of the cilium is utilized to orient the LR axis to the existing anterior-posterior (AP) and dorsal-ventral (DV) axes [1–5]. Directional ciliary beating then generates leftward flow of extraembryonic fluid [5–7], which is essential to LR development [8], and precedes other known molecular and organ asymmetries, including mesendodermal calcium [9–12], preferential degradation of *dand5* (*cerl2*, *charon*) mRNA [13] and Nodal signaling. Cilia are also believed to sense this flow [14]; however, the mechanism is unknown despite intense investigation.

One of the earliest observed molecular asymmetries is an increase in cytoplasmic mesendodermal calcium at the left side of the LRO that depends on both ciliary motility and the cation channel PKD2 and has been observed in mouse and zebrafish [9–11, 15]. Asymmetric mesendodermal calcium correlates with LR development, and is lost in *Pkd2^−/−^* mice, but the direct link to cilia is unknown. An additional connection between calcium signaling and the cilium is made by the ciliary localization of cation channels, notably PKD2. PKD2 is a six transmembrane protein that shares homology with the transient receptor potential (TRP) channels and can complex to form a voltage-sensitive, non-selective cation channel [16, 17]. Mutations in PKD2 and PKD1, which binds to and complexes with PKD2, result in human autosomal dominant polycystic kidney disease. PKD2 localizes to primary cilia in the LRO and kidney [18, 19], and mutations affecting ciliary localization of PKD2 at the LRO disrupt LR development [14, 20]. In cultured renal epithelial cells, mechanical stress on the cilium, either by direct pipette manipulation or application of laminar fluid flow, results in a rise in cytosolic calcium that depends on both PKD2 and its binding partner PKD1 [19, 21–23], suggesting that the polycystin complex can function as a mechanosensitive calcium channel. However, the in-vivo function of PKD2 at the LRO and kidney is unresolved. Further, the mechanism linking ciliary motility, sensation, polycystins, calcium and the development of molecular and morphological LR asymmetry remains a mystery.

To answer whether an intraciliary calcium signal is causative in LR development, and address whether the cilium is simply a conduit for calcium into the cell body or acts as a functional cellular calcium compartment, it is necessary to observe and manipulate calcium specifically within the cilium. To facilitate this, we and others have targeted genetically-encoded calcium indicators (GECIs) into cilia [22–24] via fusion to ciliary proteins, such as the small GTPase Arl13b [25, 26]. Cilia-targeted GECIs (GECIs) have been validated in cultured cells [22–24, 27], and this suggests that these tools may be utilized to explore the dynamics of intraciliary calcium in live embryos. Here, we simultaneously visualize and suppress intraciliary calcium in live zebrafish embryos by coupling GECIs with cilia-targeted calcium binding proteins and show that: (1) Novel intraciliary calcium oscillations (ICOs) occur along the left-side of the LRO in response to cilia motility and precede all other known molecular LR asymmetries; (2) Pkd2 initiates these ICOs to direct LR development; and (3) Intraciliary calcium signaling at the LRO is functionally required for establishing LR asymmetry.

## Results

### Intraciliary calcium responds to triptolide in cilia of cultured renal epithelial cells

**Figure 1:**
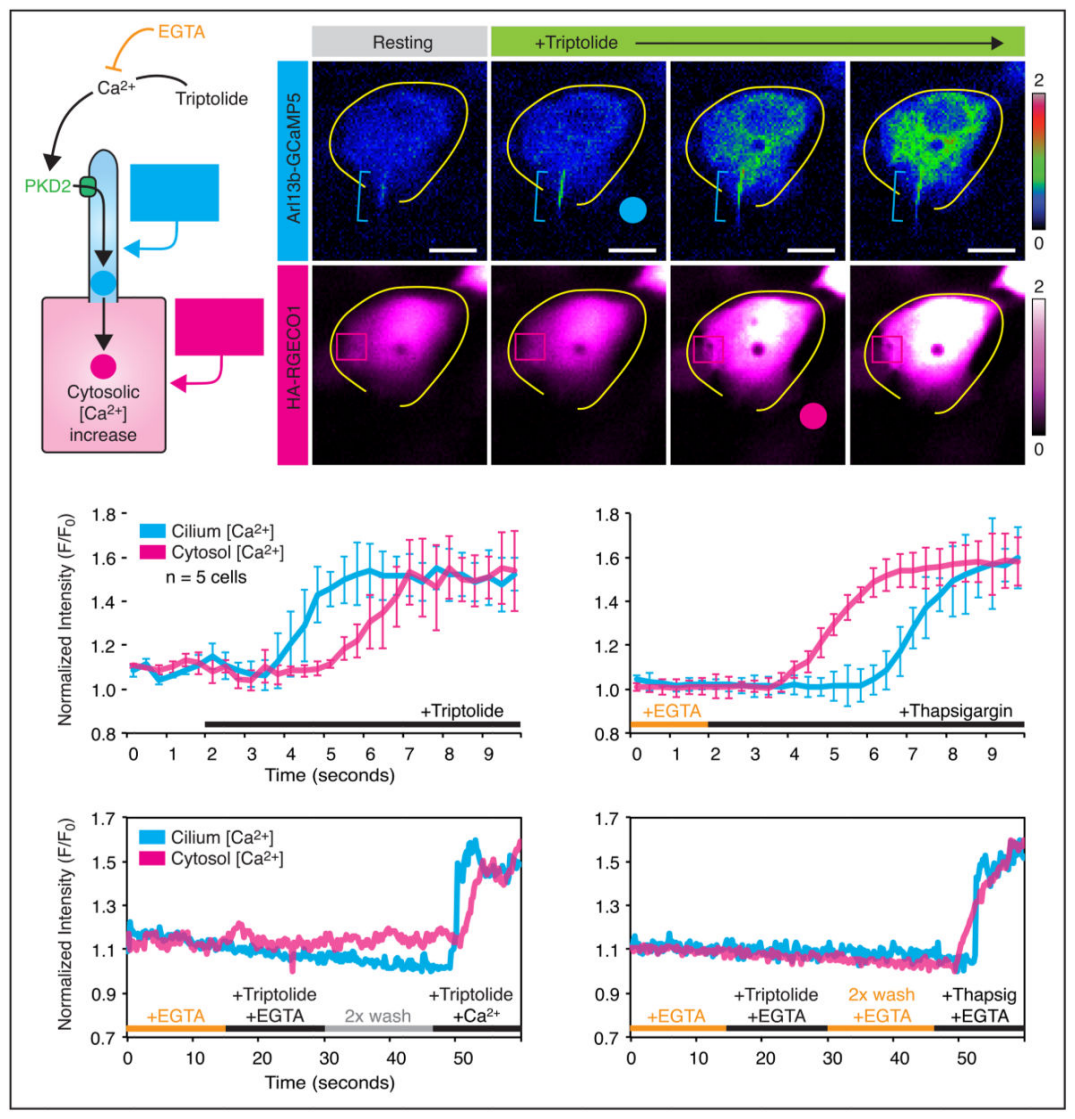
The PKD2 agonist triptolide initiates intraciliary calcium fluxes that precede cytosolic calcium waves **A.** Schematic illustrating a ciliated LLC-PK1 cell expressing *arl13b-GCaMP5* (cilium, in cyan) *and HA-RGECO1* (cytosol, in magenta). Upon triptolide treatment, a robust calcium signal can be seen throughout the cilium, which subsequently propagates into the cytosol. Residual amounts of Arl13b-GCaMP5 in the cytosol allows simultaneous visualization of intraciliary and cytosolic calcium. **B.** Representative images of a ciliated LLC-PK1 cell co-transfected with *arl13b-GCaMP5* and *HA-RGECO1* at rest and following treatment with triptolide. Cell body is outlined in yellow, cilium is identified by a cyan bracket. Scalebars = 10 μm. **C–D.** Graphs depict intensity versus time plot from the mean of five independent cell recordings of intraciliary (cyan) and cytosolic (magenta) calcium fluxes recorded in LLC-PK1 cells in response to triptolide (C) and thapsigargin (D) treatment. Traces represent mean±SEM. **E–F.** Triptolide triggered calcium fluxes require extracellular calcium. Following triptolide/EGTA treatment, subsequent addition of triptolide/Ca^2+^ (E) or thapsigargin/EGTA (F) induces sequential rise of intraciliary (cyan) and cytosolic (magenta) calcium or vice versa, respectively. Both traces are representative of three independent experimental repeats with a total of 12 cells. Duration of triptolide and thapsigargin treatment is identified by the black bar, while EGTA washes are identified in orange and Ca^2+^-free media washes in gray. All quantified intensity is shown as F/F_0_. See also Figure S1.

To simultaneously visualize calcium in the cilium and cytosol, we devised a dual reporter assay consisting of the GFP-based ciliary-GECI, GCaMP5 [28], fused to Arl13b and the RFP-based untargeted GECI, RGECO1 [29], fused to an HA tag (Fig. 1A). Both reporters responded robustly and simultaneously to treatment with the calcium ionophore ionomycin (Fig. S1A, B–C, E, G), indicating that the dual reporter system is sensitive to calcium and permits detection of distinct ciliary and cytoplasmic signals. Specificity of the system was confirmed by quenching of both signals upon addition of the permeant calcium chelator BAPTA-AM (Fig. S1D, F) and identical kinetics in response to ionomycin when both GECIs are untargeted (Fig. S1H). Strikingly, treatment with triptolide, a PKD2 agonist [30], revealed a rise of intraciliary calcium, which consistently preceded a cytosolic calcium rise by 1741 ± 235 msec (Fig. 1B–C, S1B). We then chelated extracellular calcium with EGTA followed by exposure to triptolide (Fig. 1E) or triptolide followed by thapsigargin, a SERCA inhibitor which elevates cytosolic calcium (Fig. 1F). Our results show that the mode of action of triptolide on intraciliary calcium in differentiated LLC-PK1 cells requires extracellular calcium and likely acts at the cilium based on previous localization studies [17, 18]. Intriguingly, the addition of thapsigargin elicits a response that is reverse of triptolide: cytosolic calcium elevations precede intraciliary calcium rises (Fig. 1D, S1B). Together, these results indicate that triptolide stimulates intraciliary calcium in a spatiotemporally distinct manner in cultured LLC-PK1 cells, and points to a role for polycystin2 in mediating intraciliary calcium.

### Left-biased intraciliary calcium oscillations are observed at the zebrafish left-right organizer

**Figure 2:**
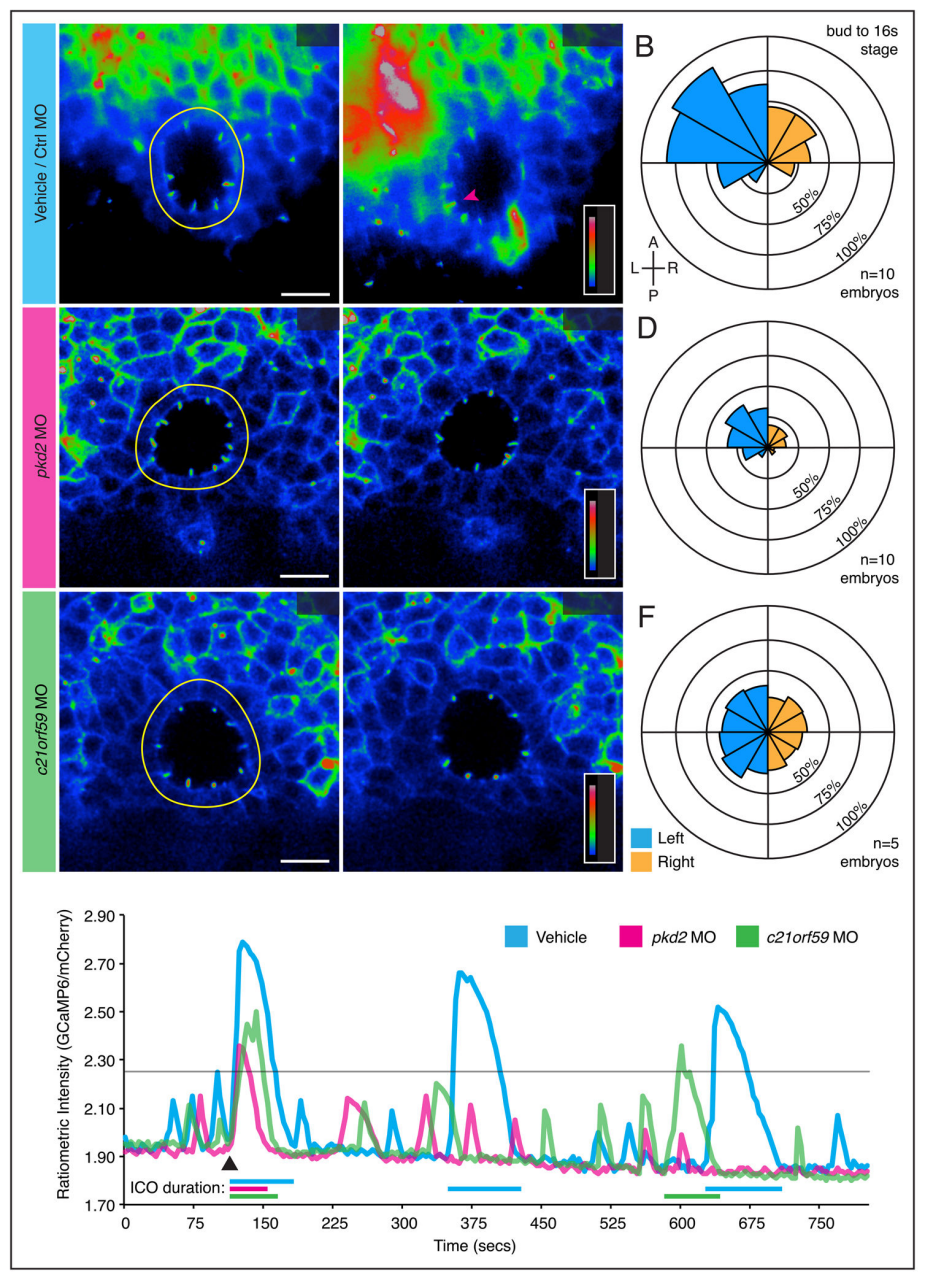
Cilia-targeted GECIs reveal the presence of ICOs in the LRO of zebrafish that are leftward-biased and dependent on Pkd2 and ciliary motility **A–F.** Spatial mapping of ICOs in the zebrafish LRO. (A, C, E) Representative fluorescent live images of LRO from vehicle-injected (A), *pkd2* morphant (MO) (C) and *c21orf59* morphant (E) zebrafish embryos at the 1-somite stage expressing ratiometric GECI (arl13b-GCaMP6/mCherry; ratiometric signal shown and false-colored with rainbow intensometric scale). White arrowheads indicate LRO cilia at resting state, while magenta arrowhead (A) highlights an ICO inducing a cytosolic calcium wave. (B, D, F) Rose diagrams depicting the spatial distribution and mean percentage of cells per embryo displaying ICOs in the total LRO of vehicle (B), *pkd2* MO (D) and *c21orf59* MO (F) embryos spanning the entire course of LRO development (bud to 16-somite stage). Rings correlate with mean percentage of cells exhibiting calcium oscillations per embryo from bud to 16-somite stage (n = 10 embryos at all stages for *pkd2* and vehicle; n = 5 embryos for *c21orf59* and vehicle). Control and experimental samples were acquired in a pair-wise manner and analysis was performed on time-lapse recordings spanning bud to 16-somite stage. Control samples for *c21orf59* morphants are not shown as the distribution and frequency was equivalent to controls for *pkd2* morphants. Left-sided calcium, blue. Right-sided calcium, orange. **G.** Intraciliary calcium at the LRO exhibits oscillation-like dynamic. Ratiometric intensity over time plot of a single cilium exhibiting ICOs in vehicle (blue), *pkd2* MO (magenta) and *c21orf59* MO (green) LROs at the 1–4 somite stage. Each plot was aligned post-acquisition to the first detected ICOs (indicated by black arrowhead) and thresholded (indicated by black horizontal line) to facilitate analysis of calcium oscillation dynamics. A: Anterior, P: Posterior, L: Left, R: Right. Scalebars in A,C,E: 10 μm. See also Figures S2 and S3; and Movies 1–3.

After cell-based validation, we applied cilia-targeted GECIs to investigate intraciliary calcium in vivo at the zebrafish LRO. We utilized Arl13b as a ciliary targeting vehicle because overexpression of *Arl13b* at doses allowing efficient visualization of the cilium does not cause a discernible LR phenotype (Fig. 5A) [31] [32] or ciliary length defects (Fig. S6F). To circumvent false-positive changes in calcium resulting from embryo thickness and positioning, we designed a ratiometric imaging strategy by co-expressing *arl13b-mCherry*, an RFP-based ciliary marker, with *arl13b-GCaMP6* by injecting in vitro synthesized mRNA into zebrafish embryos at the 1-cell stage (Fig. S2A). *Arl13b-GCaMP6* labels cilia throughout the embryo (Fig. S2A) and is easily detectable in the LRO. Remarkably, live, ratiometric-based time-lapse confocal microscopy of the LRO revealed the presence of highly dynamic intraciliary calcium oscillations (ICOs) that were previously unknown (Fig. 2A,B, S2B–D; Movie 1; shown at the 1–4 somite stage in 2A and cumulative total from bud to 16 somite stage in 2B). These oscillations displayed a periodicity ranging from 3.3 to 5.2 minutes and a long activated duration of approximately 41 ± 5.1 seconds, which is reminiscent of the low frequency and long duration characterizing cytosolic calcium oscillations in a wide range of non-excitable cells (Fig. 2G, S2E; Movie 1) [33, 34]. The zebrafish LRO is an enclosed spherical-like structure consisting of approximately 50 ciliated cells; there is no difference in cilia number across the LR axis [35]. Strikingly, within a 5 μm optical section of the LRO containing 5–6 cilia we observe oscillations in a significantly higher percentage of cilia on the left compared to the right (P=0.0001) (Fig. 2B, S2B). This asymmetry is most pronounced in the anterior part of the LRO where ICOs are found in 78% of cilia in the anterior left quadrant compared with 42% in the anterior right quadrant (Fig. 2B). This remarkable asymmetry points to a role for ICOs in LR development.

Many of the ICOs are coupled to cytoplasmic waves, and a significantly greater number of cells exhibiting coupling between intraciliary and cytosolic calcium waves localized to the left-side of the LRO (Fig. 2A, S2B, F–G; Movie 1). Additionally, ICOs located on the left-side of the LRO frequently initiated cytosolic calcium waves that spread to neighboring LRO cells and, culminate in a rapid wave of calcium on the left extending beyond the LRO (Fig. 2A, S2H, Movie 1; average number of cells affected by calcium wave: 11.9 ± 2.1). Notably, both the ICOs and the subsequent cytoplasmic calcium waves are dynamic and exhibit a similar duration. Thus, the temporal dynamics of ICOs and cytoplasmic waves are distinct from that observed in the previously reported stable elevation of calcium at later stages in the mesendoderm around the LRO [9–12] (compare Movie 1 and Movie 5). Combined with our cell-based studies, these findings support the coupling of intraciliary calcium to cytosolic calcium and indicate that intraciliary calcium signals precede cytosolic calcium at both the cellular and tissue-level.

### Intraciliary calcium oscillations require Pkd2 and ciliary motility

The activation of intraciliary calcium by triptolide that we observed in cultured cells lead us to investigate a potential role for Pkd2 in modulating intraciliary calcium levels at the LRO. We depleted *pkd2* expression in zebrafish by co-injecting a previously published translational blocking morpholino oligo against *pkd2* [36], which leads to random heart looping similar to the cardiac looping phenotype observed in *Pkd2^−/−^* mouse embryos [20, 37–41]. In *pkd2* morphants, the total number and amplitude of both ICOs and cytoplasmic calcium waves is dramatically suppressed, with a shorter duration (25 ± 2.5 sec) and longer periodicity (10.6 to 13.3 min) (Fig. 2C,D,G; S2E–G, Movie 2). Further, the dynamic spread of calcium waves across the left-side of the LRO that is preceded by ICOs along the left-side of the LRO is significantly inhibited or suppressed in *pkd2* morphants (Fig. 2C, S2H; Movie 2 average number of cells affected by calcium wave: 3.5 ± 0.9). Interestingly, however, some low-level asymmetry in the residual calcium oscillations is preserved in *pkd2* knockdown embryos (Fig. 2D), consistent with the observation that ciliary motility is normal in the absence of functional Pkd2 (Fig. S2I,J) [14, 42, 43]. To further validate the *pkd2* morpholino result, we analyzed ICOs in *pkd2^−/−^* zebrafish embryos [36], and observed a similar suppression of ICOs (Fig. S3 A–F). Longitudinal evaluation of the imaged embryos demonstrated random heart looping in the *pkd2^−/−^* embryos, and normal heart looping in the control siblings (Fig. S3G). Coupled with recent cell-based observations that intraciliary calcium can be triggered by artificial flow [22, 23], these data suggest that Pkd2 at the LRO cilium acts as part of a calcium channel, and that Pkd2 initiates ICOs to direct asymmetric cytoplasmic calcium signaling during LR development.

**Figure 3:**
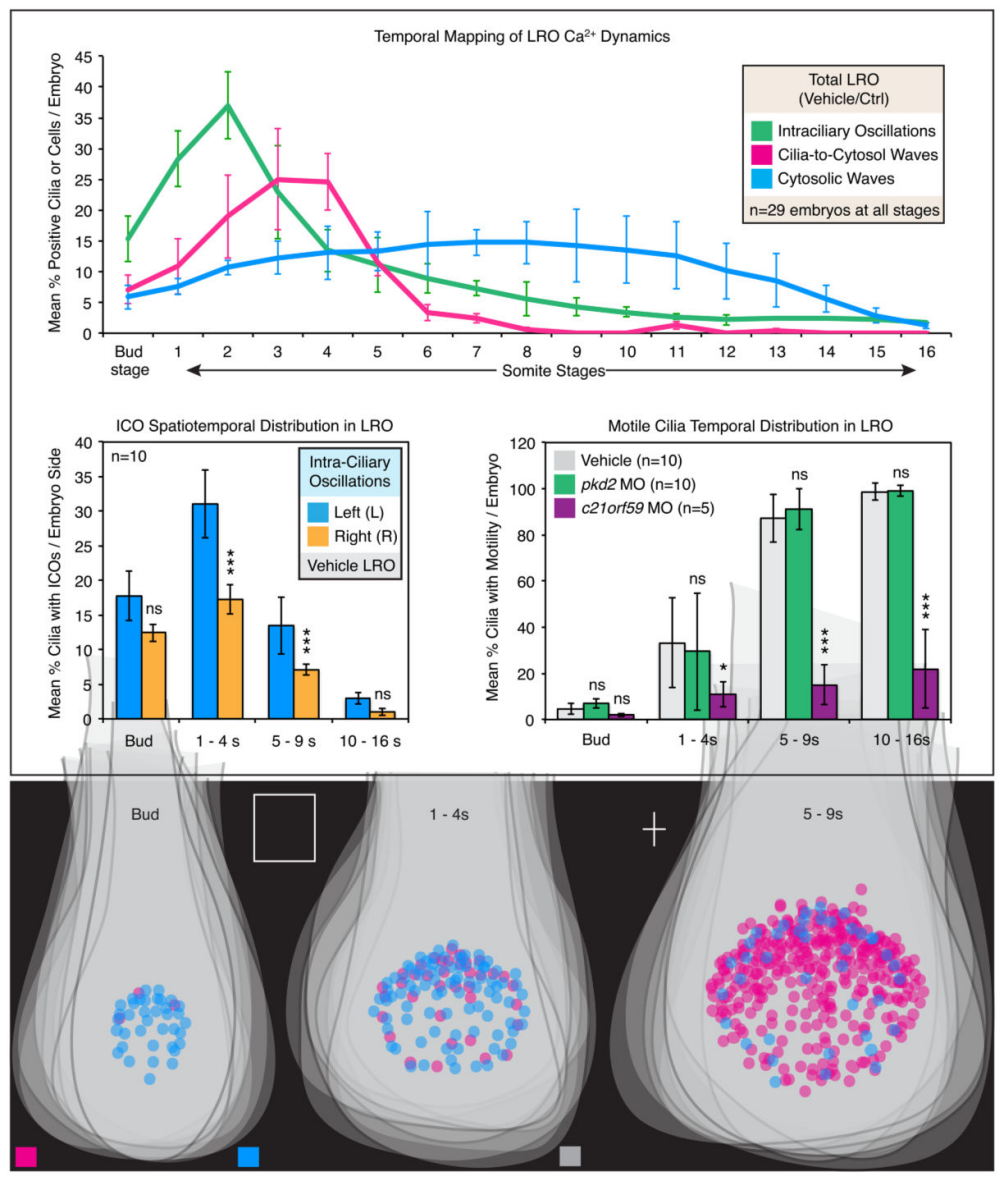
ICOs are spatiotemporally asymmetric and coincide with the initiation of cilia motility in the LRO **A.** Temporal mapping of mean percentage of cells exhibiting calcium activity in vehicle LRO (n=29 embryos) from bud-stage to 16-somite-stage. Calcium activity in the LRO was categorized as ICOs (green), cilia-to-cytosolic waves (magenta) and cytosolic waves (cyan). **B.** Spatiotemporal mapping of mean percentage of cells per embryo exhibiting ICOs on the left or right-side of the LRO in vehicle embryos (n=10 embryos) during bud, 1–4 somite, 5–9 somite and 10–16 somite stages. Left-sided calcium oscillations = blue; right-sided calcium oscillations = orange. **C–D**. Spatiotemporal mapping of two cilia populations in the zebrafish LRO. (C) Temporal mapping of mean percentage of motile cilia in the vehicle (gray), *pkd2* MO (green) and *c21orf59* MO (violet) LROs over the course of LRO development. (D) Additive projections of motile (magenta) and immotile (cyan) cilia from vehicle embryos over the course of LRO development. Tailbud area and morphology (gray) of corresponding LROs are also assembled by additive projections. A: Anterior, P: Posterior, L: Left, R: Right. All data shown is mean±SEM. See also Figure S4.

To directly link ciliary motility and ICOs at the LRO, we paralyzed ciliary motility in the LRO by morpholino knockdown of *c21orf5*9, which encodes a component required for dynein arm assembly in motile cilia and is necessary for proper LR patterning in humans and zebrafish [44]. At the 6–8 somite stage, control embryos displayed a LRO ciliary beat frequency of a 45.4 ± 6.8 Hz, with 87.1% of all cilia displaying motility (Fig. 3C,D, S2I–J). In contrast, *c21orf59* knockdown embryos displayed a mean ciliary beat frequency of 2.3 ± 3.3 Hz with only 15.0% of all cilia displaying any motility (Fig. 3C, S2I–J). Further, *c21orf5*9 knockdown embryos display loss of directional fluid flow, thus supporting that *c21orf59* lack functional motile cilia [44]. In *c21orf59* knockdown fish, ICOs are suppressed in abundance, duration (29 ± 6.6 sec), periodicity (ranging from 6.5 to 9.6 min) and amplitude (Fig. 2E–G, S2E, Movie 3). Further, the normal left-bias of ICOs and cytoplasmic calcium waves is not observed (Fig. 2E,F; Fig. S2G; Movie 3). Further, the spread of calcium waves across the left-side of the LRO is significantly decreased in *c21orf59* knockdown embryos (Fig. 2E, S2F–H; average number of cells affected by calcium wave in *c21orf59:* 6.6 ± 2.9). These findings indicate that LRO cilia respond to ciliary motility by inducing Pkd2-dependent ICOs along the left-side of the LRO.

### Intraciliary calcium oscillations coincide with the onset of left-right organizer cilia motility and are the earliest molecular left-right asymmetry

We then investigated the temporal progression of the calcium dynamics in relation to known LR molecular asymmetries. We observed that Pkd2 and ciliary motilty-dependent ICOs at the LRO, both total and left-sided, peaked during early LRO stages (1 to 4-somite stages) and diminished at later stages (5 to 16-somite stages) (Fig. 3A,B, S4A,B), much earlier than anticipated. In addition to ICOs, we also detected asymmetric cytosolic calcium waves at the LRO that peaked at later stages (5 to 9-somite stages), consistent with a recent report [15] (Fig. 3A, S4D,E). The coupling of intraciliary calcium to cytosolic calcium was even more strongly biased toward the left side of the LRO and also strongly peaked at the 1 to 4-somite stages (Fig. 3A, S4C), suggestive of an additive mechanism for transducing intraciliary calcium. The earliest reported asymmetric molecular cue following fluid flow in the LRO has been asymmetric expression of *Dand5* in the mouse and frog LRO; the zebrafish homologue of *Dand5 (charon)* displays asymmetric expression at the LRO beginning at the 8–10 somite stage [45], well after asymmetric ICOs. Thus, our findings implicate dynamic, LR asymmetric calcium oscillations within cilia of the LRO at 1–4 somite stages as the earliest known asymmetric signaling event during LR development.

To define the temporal relationship between ICOs and LRO ciliary motility, we used the ciliary marker Arl13b-mCherry to spatio-temporally map motile and immotile cilia populations across LRO development. At the end of gastrulation, the zebrafish LRO forms and gradually becomes ciliated. We observed that at the earliest stage (bud stage), LRO cilia are primarily immotile, with only 4.6 ± 2.3% of all cilia exhibiting motility (Fig. 3C,D). The percentage of cilia exhibiting motility rapidly increases to 33.3 ± 19.6% at the 1–4 somite stage; indicating that this is the developmental window at which cilia motility and likely early fluid flow is triggered in the zebrafish LRO. Notably this coincides with the onset of ICOs (Fig. 3A,B, Fig. S4A). At subsequent stages, nearly all LRO cilia exhibit motility, representing 98.7 ± 3.6% of total cilia in the LRO by the 10 to 16-somite stages. Our data show that ICOs coincide with the onset of cilia motility at the LRO, couple with cytoplasmic calcium waves and are the earliest asymmetric LR molecular cue identified thus far.

To investigate the effect of cilia paralysis on the temporal progression of LRO ICOs, we examined *c21orf59* knockdown embryos over the course of LRO development. Notably, in contrast to *pkd2* knockdown, the baseline ICOs observed at the bud stage are unaffected by ciliary paralysis, even though the normal increase in left-sided oscillations coinciding with ciliary motility is lost (Fig. S4A,B). In the presence of Pkd2, but the absence of ciliary motility, cilia are still competent to receive signals potentially generated by random perturbations while ciliary motility is required to bias the signal to the left. Notably, the LR randomization of ICOs observed in the *c21orf59* knockdown embryos reflects the randomized organ situs phenotype observed in mice and humans with mutations resulting in ciliary paralysis [46].

### Intraciliary calcium oscillations are upstream of left-right asymmetrical mesendodermal calcium

**Figure 4:**
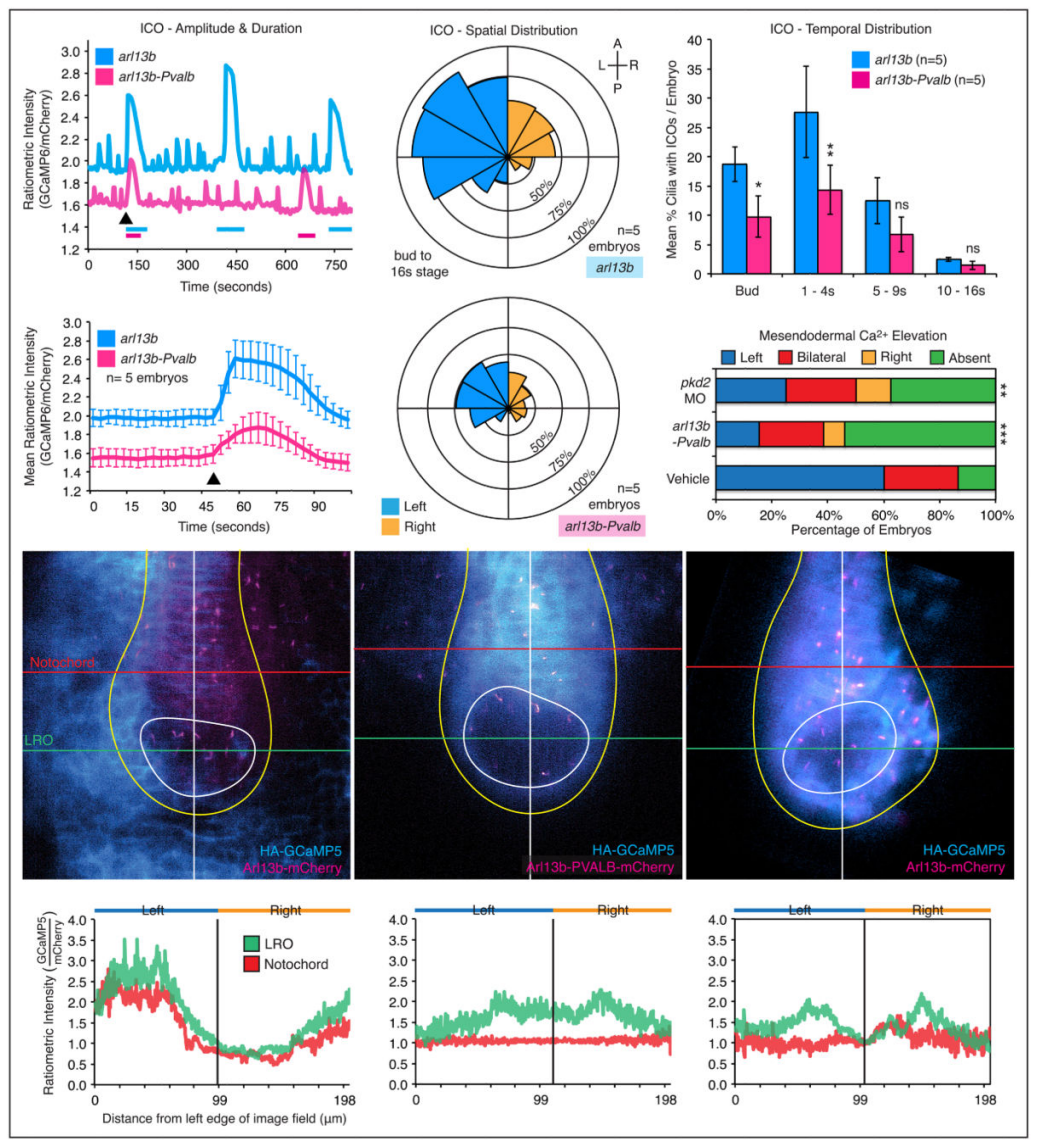
ICOs are required for establishing asymmetric mesendodermal calcium at the LRO **A–D.** Suppression of ICOs by *arl13b-Pvalb*. (A–B) Ratiometric intensity (arl13b-GCaMP6/mCherry) over time plot of a single cilium from a representative embryo (A) or a mean trace representing five embryos (B) exhibiting ICOs in *arl13b* (cyan) and *arl13b-Pvalb* (magenta) LROs at the 1–4 somite stage. Each plot was aligned post-acquisition to the first detected ICOs (indicated by black arrowhead) and thresholded to facilitate analysis of calcium oscillation dynamics. (C) Rose diagrams depicting the spatial distribution and mean percentage of cells per embryo displaying ICOs in the total LRO of *arl13b* (upper) and *arl13b-Pvalb* (lower) embryos spanning the entire course of LRO development (bud to 16-somite stage). Rings correlate with mean percentage of cells exhibiting calcium oscillations per embryo from bud to 16-somite stage (n = 5 embryos at all stages for *arl13b* and *arl13b-Pvalb*). Control and experimental samples were acquired in a pair-wise manner and analysis was performed on time-lapse recordings spanning bud to 16-somite stage. Left-sided calcium, blue. Right-sided calcium, orange. (D) Temporal mapping of mean percentage of cells exhibiting ICOs per embryo in the entire LRO in *arl13b* (cyan) and *arl13b-Pvalb* (magenta) embryos across the entire course of LRO development (bud to 16-somite). Data is shown as mean±SEM. **E–G.** Disruption of mesendodermal calcium by *arl13b-Pvalb*. (E) Distribution of vehicle, *arl13b-Pvalb* and *pkd2* MO embryos with normal left, or abnormal right, bilateral and absent mesendodermal calcium signal at the LRO. *pkd2*: **P=0.0033; *arl13b-Pvalb*: ***P=0.0002. (F) Representative live fluorescent images depicting cytoplasmic calcium in mesendodermal cells of vehicle, *arl13b-Pvalb-mCherry* and *pkd2* MO zebrafish embryos at the 6 to 8-somite stage. Calcium is visualized with HA-GCaMP5 (cyan) and cilia are identified by Arl13b-mCherry (magenta). Scalebars = 30 μm. A: Anterior, P: Posterior, L: Left, R: Right. (G) Ratiometric intensity plot (GCaMP5/mCherry) of cytoplasmic mesendodermal calcium levels corresponding to images in F, relative to position at the LRO level (green) and posterior notochord level (red) across the left-right axis. See also Figures S5, and Movies 4–6.

To directly test whether intraciliary calcium is correlative or causative in breaking LR symmetry, we developed a method to specifically suppress intraciliary calcium. The calcium binding protein (CBP) Parvalbumin (PVALB) has been used effectively as a calcium sink to suppress cytoplasmic and nuclear calcium signaling [47–49]. We fused PVALB with Arl13b, and the fluorophore mCherry as a visual marker. Coexpression with the intraciliary calcium indicator *arl13b-GCaMP6* in zebrafish (Fig. S5A) revealed that *arl13b-Pvalb-mCherry* suppresses both resting intraciliary calcium, and the abundance, amplitude and periodicity (ranging from 5.6 to 8.9 min) of ICOs (Fig. 4A–D, S5B–D, Movie 4).

Having demonstrated that Arl13b-PVALB effectively inhibits intraciliary calcium, we then utilized it to address whether asymmetric intraciliary calcium is specifically required for establishing proper lateralization of known LR asymmetrical molecular markers. We first analyzed the effect of intraciliary calcium on the previously observed stable-level of mesendodermal calcium along the left-side of the LRO [9–11, 15] [12]. Live imaging of the LRO in intact wildtype embryos co-expressing *arl13b-mCherry* and *HA-GCaMP5* revealed the presence of a robust, stable elevation in mesendodermal calcium in a broad domain extending from the left of the LRO during a 2 hour window extending from the 6-somite stage to the 10-somite stage in 60% of observed embryos (total n = 15 embryos, Fig. 4E–G; Movie 5, 6). Stable mesendodermal calcium is temporally distinct from ICOs and the associated transient cytoplasmic calcium waves at the left of the LRO that is observed at the 1–4 somite stage. Notably, this asymmetric elevation in mesendodermal calcium also encompasses the left-side of the posterior notochord, thus displaying an asymmetric pattern which is broader than previously documented [12] [9]. This is presumably because of the sensitive nature or delivery efficiency of GCaMP5/6 relative to the calcium dye Fluo3, which is loaded by whole-embryo bathing. In contrast, embryos co-expressing *arl13b-Pvalb-mCherry* and *HA-GCaMP5* displayed a striking loss of stable asymmetric mesendodermal calcium at the LRO and posterior notochord: only 15.4% of observed embryos displayed asymmetry (total n = 13 embryos, Fig. 4E–G; Movie 6). Importantly, the effect of Arl13b-PVALB-mCherry on mesendodermal calcium was specific to the cilium, as asymmetric mesendodermal calcium was not disturbed in embryos with an equimolar dose of HA-PVALB-mCherry in the cytoplasm (Fig. S6A–D). To rule out that the effect observed from *arl13b-Pvalb-mCherry* resulted from residual cytosolic Pvalb, we quantified the mCherry signal from *arl13b-Pvalb-mCherry* embryos and showed a lower relative amount of PVALB in the cytoplasm when compared to embryos expressing *HA-Pvalb-mCherry*. (Fig. S5E–G). Combined with the predicted volume of the cilium, which is approximately 1/10,000 of the cytosol [23], this predicts a fusion protein concentration enrichment of 10^4^ in the cilium relative to the cytosol. Similar to inhibition of intraciliary calcium by *arl13b-Pvalb*, morpholino knockdown of *pkd2* also resulted in loss of asymmetric mesendodermal calcium (Fig. 4E–G; Movie 6). Together, these results demonstrate that intraciliary calcium is upstream of asymmetric mesendodermal calcium at the LRO and that Pkd2 modulates intraciliary calcium to affect LR mesendodermal calcium asymmetry.

### ICOs direct organ laterality and Nodal asymmetry in an LRO-autonomous manner

**Figure 5:**
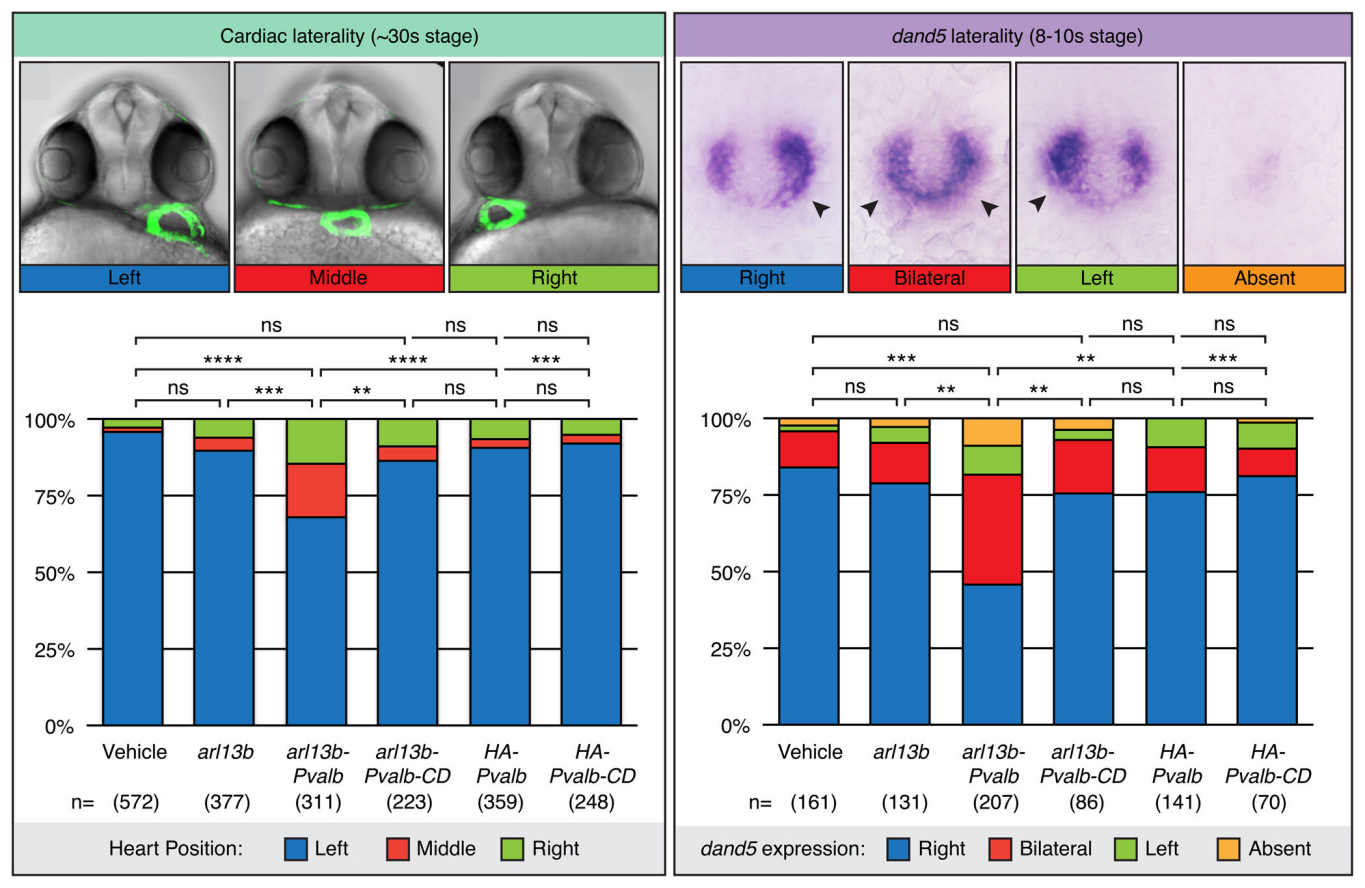
Intraciliary calcium is essential for vertebrate LR development **A.** Representative brightfield and fluorescent images showing heart position in zebrafish embryos at ~30-somite stage highlighted by a GFP transgene driven by the promoter of *cardiac myosin light chain 2* (*cmlc2*). The embryo is seen from the ventral side, showing normal left (blue), and abnormal middle (red) and right (green) heart loops. Graph shows distribution of heart position in response to expression of parvalbumin (PVALB) and mutant parvalbumin (PVALB-CD) targeted to cilia or cytoplasm of wildtype embryos. Data shown is pooled from nine total independent experiments. **B.** Representative images of whole-mount *in situ* hybridization for *dand5 (charon)* expression in the LRO of zebrafish embryos at the 8–10 somite stage. The embryo is seen from the dorsal side, showing normal right-sided (blue) expression and abnormal bilateral (red), left-sided (green) or absent (orange) expression. Graph shows distribution of *dand5* in response to expression of PVALB and mutant parvalbumin (PVALB-CD) targeted to cilia or cytoplasm. Data shown is pooled from three independent experiments. Statistical comparison was analyzed by one-way ANOVA with Tukey’s multiple comparison test. *P < 0.05, while ** *P* < 0.005, ***P < 0.0005, respectively. NS (not significant): *P* ≥ 0.05. Asterisks above brackets denote comparisons between samples indicated by the bracket, while asterisks above a single sample denote comparisons between that sample and the control. n = Total number of embryos analyzed for each experimental condition (in parentheses). See also Figure S6.

We then directly tested the functional relevance of intraciliary calcium for the development of organ LR asymmetry. 31.8% of *arl13b-Pvalb-mCherry* embryos displayed LR defects as indicated by heart positioning (Fig. 5A). Notably, no significant LR defects were observed in vehicle-injected embryos or embryos over-expressing an equimolar dose of *arl13b* alone (Fig. 5A). To determine the developmental stage at which Arl13b-PVALB interferes with LR asymmetry, we analyzed the expression domain of *southpaw (spaw)*, a *Nodal* homolog that is normally expressed in the left lateral plate mesoderm [50], and *dand5* (*charon*), an upstream repressor of *spaw* [51]. A significant number of *arl13b-Pvalb* injected embryos displayed bilateral, absent or left-sided expression of *dand5* (54.1%) (Fig. 5B) and bilateral, right or absent *spaw* (46.7%), compared with control groups (Fig. S5H), demonstrating that ciliary calcium signaling is required upstream from the asymmetrical expression of *dand5*.

To definitively rule out the possibility that residual cytoplasmic Arl13b-PVALB-mCherry is responsible for the LR defect in *arl13b-Pvalb-mCherry* expressing embryos, we expressed the untargeted *HA-Pvalb-mCherry* at an equimolar dose as *arl13b-Pvalb-mCherry* (Fig. S5E–G) and found it did not generate significant LR defects (Fig. 5A,B; Fig. S5H), thereby suggesting that the LR defects observed in *arl13b-Pvalb-mCherry* expressing embryos are specific to the cilium. Notably, significantly larger doses of *HA-Pvalb-mCherry* do suppress cytosolic calcium and yield LR defects (Fig. S6E), consistent with a role for cytosolic calcium in regulating LR asymmetry at downstream steps. To ascertain that the LR defects are specific to the calcium-binding function of Arl13b-PVALB, we delivered Arl13b-PVALB-CD [48], which contains a mutation in the calcium binding domain that reduces its affinity for calcium, to the cilium. The Arl13b-PVALB with the CD mutation did not result in LR defects relative to the wildtype Arl13b-PVALB (Fig. 5A,B; Fig. S5H).

As calcium can alter ciliary motility in unicellular organisms, we analyzed beat frequency of LRO cilia and found no effect of *arl13b-Pvalb-mCherry* expressed at doses that significantly affect LR development (Fig. S6H). LRO size and morphology was also not affected (Fig. S6G). Although the lack of any observable effect of Arl13b-PVALB on LRO cilia motility was somewhat surprising, reduction in intraciliary calcium induced by Arl13b-PVALB could be insufficient to affect ciliary motility. Further, application of ionomycin or thapsigargin did not alter ciliary motility or fluid flow in the mouse LRO; thereby suggesting that LRO ciliary motility may be independent of calcium [15].

**Figure 6:**
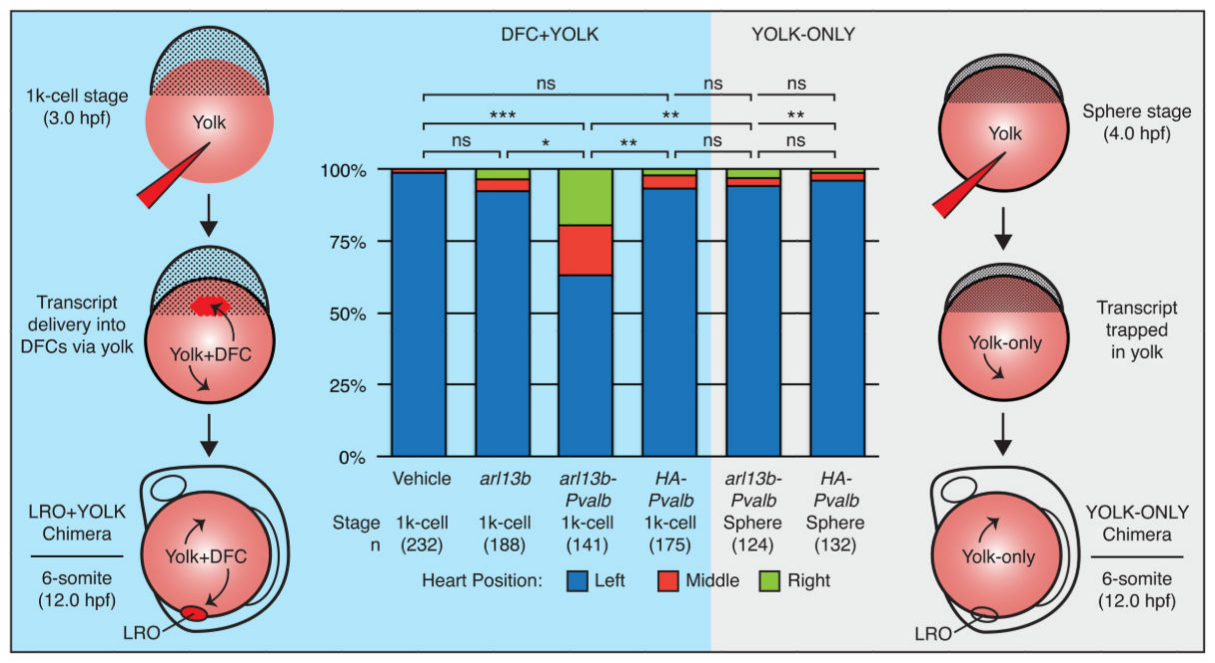
Intraciliary calcium is autonomously required at the LRO for proper LR patterning **A–C**. The LR defects induced by *arl13b-Pvalb* are LRO autonomous. Microinjection into the yolk at 3.0 hpf (A) facilitates transcript delivery into the dorsal forerunner cells (DFCs, LRO precursor cells in zebrafish), while microinjection into the yolk at 4.0 hpf (B) traps transcripts in the yolk-only (control). (C) Graph shows distribution of heart looping defects resulting from DFC-specific expression of *arl13b-Pvalb*. Data shown pooled from three total independent experiments. Statistical comparison was analyzed by one-way ANOVA with Tukey’s multiple comparison test. *P < 0.05, while ** *P* < 0.005, ***P < 0.0005, respectively. NS (not significant): *P* ≥ 0.05. Asterisks above brackets denote comparisons between samples indicated by the bracket, while asterisks above a single sample denote comparisons between that sample and the control. n = Total number of embryos analyzed for each experimental condition (in parentheses).

To demonstrate that intraciliary calcium is required at the level of the LRO, we generated chimeric zebrafish embryos with *arl13b-Pvalb* specifically expressed in LRO cells using a well-established method (Fig. 6A,B) [4] [52]. LRO-specific expression of the cilia-targeted calcium suppressor *arl13b-Pvalb* yielded significant laterality defects (36.4%), while yolk alone expression, and expression of control constructs had no impact on LR development (Fig. 6C). Combined, these results show a LRO autonomous requirement for intraciliary calcium signaling in establishing LR asymmetry.

## Discussion

In this study, we identify *in vivo* ICOs as the earliest known LR asymmetric signaling cue, coinciding with the onset of ciliary motility at the LRO. Most importantly we show that intraciliary calcium signaling depends on Pkd2 and ciliary motility and is essential for the development of LR asymmetry. Based on these findings, we propose a model for the development of LR asymmetry in which motile cilia in the LRO that trigger Pkd2-dependent ICOs along the left-side of the LRO (outlined in Fig. S6I and the graphical abstract). ICOs are then transduced and relayed into the cytosol and trigger a secondary wave of calcium in the mesendodermal cells surrounding the left-side of the LRO, subsequently inducing components of the *Nodal* signaling cascade to establish LR asymmetry.

Our findings that ICOs peak and accumulate along the left-side of the LRO with the onset of cilia motility at the 1 to 4-somite stage is consistent with a role for intraciliary calcium in initiating sensory response to ciliary motilty at the LRO. The observation that intraciliary calcium waves peak earlier than cytosolic waves raise the possibility that cumulative waves of intraciliary calcium are required to establish a robust cytosolic calcium signal across the left side of the LRO. It is interesting to speculate that a succession of calcium waves could be generated locally, and thereby support observations that directional flow generated very locally by as few as two motile cilia at the LRO can establish normal LR asymmetry [53]. An intriguing interpretation of this finding is that the frequency of intraciliary calcium waves is initially determined by the number of immotile sensory cilia, which are more abundant at earlier timepoints, while robust asymmetry is established and amplified by stronger directional flow created by increasing numbers of motile cilia at later timepoints. The nature of the signal that triggers asymmetric ciliary signals at the LRO remains an active area of investigation [9, 11]. Since early ICOs are Pkd2-dependent, and polycystins are thought to function as mechanosensors, our data supports a mechanical signal causing early asymmetric ICOs. It remains possible that interaction of mechanical and chemical signals is required for robust left-right asymmetry.

Many features of the cilium make it uniquely able to provide a discrete calcium compartment for sensing ciliary motility, including its small size, large surface area and membrane configuration. The ciliary membrane is contiguous with the cytoplasmic membrane and there is direct passage between cilia and cytoplasm at the ciliary base. By contrast, most organelles are separated from the cytoplasm by enclosing membranes. It is possible that the exceedingly large ciliary membrane to volume ratio contributes to the difference between intraciliary and cytoplasmic calcium, theoretically creating greater influx to efflux. Notably, Pkd2 is also localized in the ER. It is possible that Pkd2 at the cilium initiates ICOs, while Pkd2 in the ER is involved in amplifying the small ciliary calcium signal to a large cytoplasmic calcium signal. However, it remains to be seen whether there is also a biological barrier that controls the interrelationship between intraciliary and cytoplasmic calcium. For example, transition zone components, nucleoporins at the cilium [54] and the calmodulin-binding protein Inversin [55] are attractive candidates as intraciliary calcium gatekeepers.

Given the diverse roles of PKD2 in a wide range of biological functions, such as renal homeostasis, mating behavior, sperm motility and sour taste [56] [57] [58, 59], the Pkd2-dependent ICOs identified here may have broader implications for cilia mediated signaling. It has been shown that the PKD1L1-PKD2L1 complex functions as a ciliary calcium channel in retinal pigmented epithelial cells (hTERT-RPE1) [27]. In addition, the temporal delay between ciliary and cytoplasmic calcium in response to a stimulus differs between hTERT-RPE1 retina cells [24, 27] and the LLC-PK1 kidney cells shown here. Together these observations suggest the existence of tissue-specific ciliary calcium channels and dynamics.

The ICOs we observed at onset of ciliary motility in the LRO are fleeting. Compared to calcium signals observed in excitable tissues, the LRO ICOs are long in duration and low in frequency. However, their frequency is comparable with cytoplasmic calcium flashes observed in crown cells of the mouse LRO at the equivalent stage [15], and is comparable to cytosolic calcium oscillations observed in a number of non-excitable tissues [34, 60–62]. The molecular mechanisms coupling ICOs to cytoplasmic calcium signaling and LR asymmetry remain unclear. Interestingly, in cultured cells, such oscillations were able to induce changes in gene expression [60]. We show that when ICOs are suppressed, *dand5* is not downregulated on the left-side of the LRO in response to ciliary motility. Since it has been shown that downregulation of *dand5* is accomplished by degradation of *dand5* mRNA via the 3′ UTR at the left of the LRO [13], an intriguing hypothesis is that *dand5* mRNA degradation is regulated by miRNAs in a calcium dependent manner. This could suggest a role for the RNA-binding protein BICC1, which is associated with defective LR development when knocked out in mice [63].

Beyond the essential role for intraciliary calcium in LR development, cilia, fluid flow and calcium are central to many developmental processes and human diseases. The approach we show here for real-time, live study of LR development in intact vertebrate embryos opens a window into the potentially far-reaching role of intraciliary calcium in development.

## Experimental Procedures

### Microscopy

All live imaging was performed on an inverted 710 DUO/NLO (Zeiss) microscope with a 40x or 63x water C-apochromat objective, a 7-LIVE scanner with multiple high-speed CCD line detectors, a 488 nm laser, a 561 nm laser, and a 633 nm laser. Zen 2009 (Zeiss) was used for acquisition, ImageJ (NIH) and Volocity (PerkinElmer) were utilized for post-acquisition analysis, and Final Cut Pro (Apple) was used for video editing and preparation.

### Live imaging of zebrafish LRO

Knockdown and overexpression studies were performed in zebrafish by microinjection of morpholinos or in vitro transcribed mRNA at the 1-cell stage. Live imaging of the LRO was performed as previously described [64]. Briefly, embryos were microinjected at the 1-cell stage, then cultured at 28.5°C until the stage of interest. After dechorionation, embryos were submerged in 1% low melt agarose and mounted into 8-well coverglass-bottom slides (Nunc), with the tailbud mounted down towards the coverglass. The agar block was then covered with a drop of standard embryo medium and the embryos were then imaged at the indicated stages of interest.

### Live imaging of cultured renal cells

LLC-PK1 cells were cultured in DMEM:F12 growth medium containing 5% serum and incubated at 37°C with 5% CO_2_. Cells were grown to 70% confluency onto coverglass, transfected utilizing Lipofectamine 2000 (Invitrogen) and then starved for 48 hours to induce ciliogenesis. During live imaging, cells were mounted and incubated on an inverted stage fitted with an environmental chamber (Zeiss) that maintained conditions at 37°C with 5% CO_2_ throughout the experiment. For pharmacological manipulations, media containing the vehicle (0.5% DMSO), 1 μM ionomycin (Sigma), 10 μM BAPTA-AM (Invitrogen), 0.1 μM triptolide (Calbiochem) or 5 μM thapsigargin (Sigma) was added to the perfusion chamber via a standard gravity input and output setup during continuous imaging.

### Statistics

*P*-values were derived from one-way ANOVA analysis and an appropriate post-hoc multi-comparison test using SPSS (IBM Corporation) in experiments with more than 2 means. Student’s t-test (two-tailed) was performed using SPSS in experiments comparing only 2 means. For spatiotemporal analysis of ICOs, ciliary-to-cytosolic waves and cytosolic waves (Figures 3B and S4B,E), chi-squared test was utilized to compare observed versus expected frequencies using SPSS. In all datasets and figures, the criteria for statistical significance was defined as *P* < 0.05. Data that did not meet these criteria (*P* ≥ 0.05) was denoted as not statistically significant (“ns”). Single asterisk indicates a comparison that results in *P* < 0.05, while double asterisk and triple asterisk indicate *P* < 0.005 and *P* < 0.0005, respectively. Asterisks above brackets denote comparisons between samples indicated by the bracket, while asterisks above a single sample denote comparisons between that sample and the control. All quantified zebrafish data represents mean ± standard error of the mean (SEM) per embryo.

Extended details and procedures, as well as morpholino sequences and additional reagents, are included in supplemental information.

## Supplementary Material

1

2

3

4

5

6

7

8

## Highlights

- LRO intraciliary calcium oscillations are the earliest molecular LR asymmetry
- Left-biased LRO intraciliary calcium oscillations depend on Pkd2 and cilia motility
- Intraciliary calcium oscillations are required for asymmetric Nodal signaling
- Intraciliary calcium is essential for the development of vertebrate LR asymmetry
