# Supplementary material for: Intraciliary calcium oscillations initiate vertebrate left-right asymmetry
Source: Curr Biol. Author manuscript; Available in PMC 2016 Mar 2. (PMC4469357; doi:10.1016/j.cub.2014.12.051)
Supplement: 1 [file NIHMS661880-supplement-1.pdf]

Intraciliary calcium initiates left-right asymmetry

S. Yuan, L. Zhao, M. Brueckner and Z. Sun


| Inventory of Supplemental Information | Page |
|---|---|
| Figure S1: Pharmacological manipulation of intra-ciliary calcium | 2-3 |
| Figure S2: Ratiometric imaging of ICOs and cytosolic calcium waves in the LRO | 4-6 |
| Figure S3: ICOs are suppressed in *pkd2* mutants | 7-9 |
| Figure S4: ICOs precede cytosolic calcium waves during LRO development | 10-11 |
| Figure S5: Suppression of intraciliary calcium by *arl13b-Pvalb* and equimolar expression relative to *HA-Pvalb* | 12-14 |
| Figure S6: Intraciliary calcium is upstream of mesendodermal calcium, LRO size and cilia motility are not affected by Arl13b-Pvalb | 15-17 |
| Movie S1: ICOs are biased to the left-side of the LRO | 18 |
| Movie S2: *pkd2* morphants exhibit a loss of ICOs at the LRO | 18-19 |
| Movie S3: *c21orf59* morphants exhibit a loss of ICOs at the LRO | 19 |
| Movie S4: *arl13b-Pvalb* mRNA expressing embryos exhibit dampening of ICOs at the LRO | 20 |
| Movie S5: Left-sided mesendodermal calcium in control embryo | 20 |
| Movie S6: Loss of asymmetrical mesendodermal calcium in *arl13b-Pvalb and Pkd2 morphant embryos* | 21 |
| Methods: Animal care ethics | 22 |
| Methods: Zebrafish husbandry and microinjection and Morpholinos | 22 |
| Methods: Cloning of GECIs, PVALB and mRNA transcription | 23 |
| Methods: Cell culture, transfection and live imaging | 23-24 |
| Methods: Calcium imaging and pharmacological manipulations | 24-25 |
| Methods: Zebrafish cardiac laterality analysis | 25-26 |
| Methods: Generation of chimeric DFC embryos | 26 |
| Methods: In situ hybridization | 27 |
| Methods: In-vivo imaging of intraciliary calcium oscillations in the zebrafish LRO | 27-30 |
| Methods: Longitudinal analysis of intraciliary calcium oscillations and cardiac laterality in *pkd2* mutant zebrafish | 30-31 |
| Methods: Spatial analysis of motile and immotile cilia | 31-32 |
| Methods: Suppression of intraciliary calcium in zebrafish LRO | 32-33 |
| Methods: Mesendodermal calcium imaging and cilia motility in the zebrafish LRO | 33-34 |
| Methods: Arl13b cilia length measurements in the LRO | 34-35 |
| Methods: Preparation of embryo lysates and western blots | 35 |
| Methods: Statistics | 36 |
| Supplementary References | 37-38 |
