# Supplementary material for: Intraciliary calcium oscillations initiate vertebrate left-right asymmetry
Source: Curr Biol. Author manuscript; Available in PMC 2016 Mar 2. (PMC4469357; doi:10.1016/j.cub.2014.12.051)
Supplement: 2 [file NIHMS661880-supplement-2.pdf]

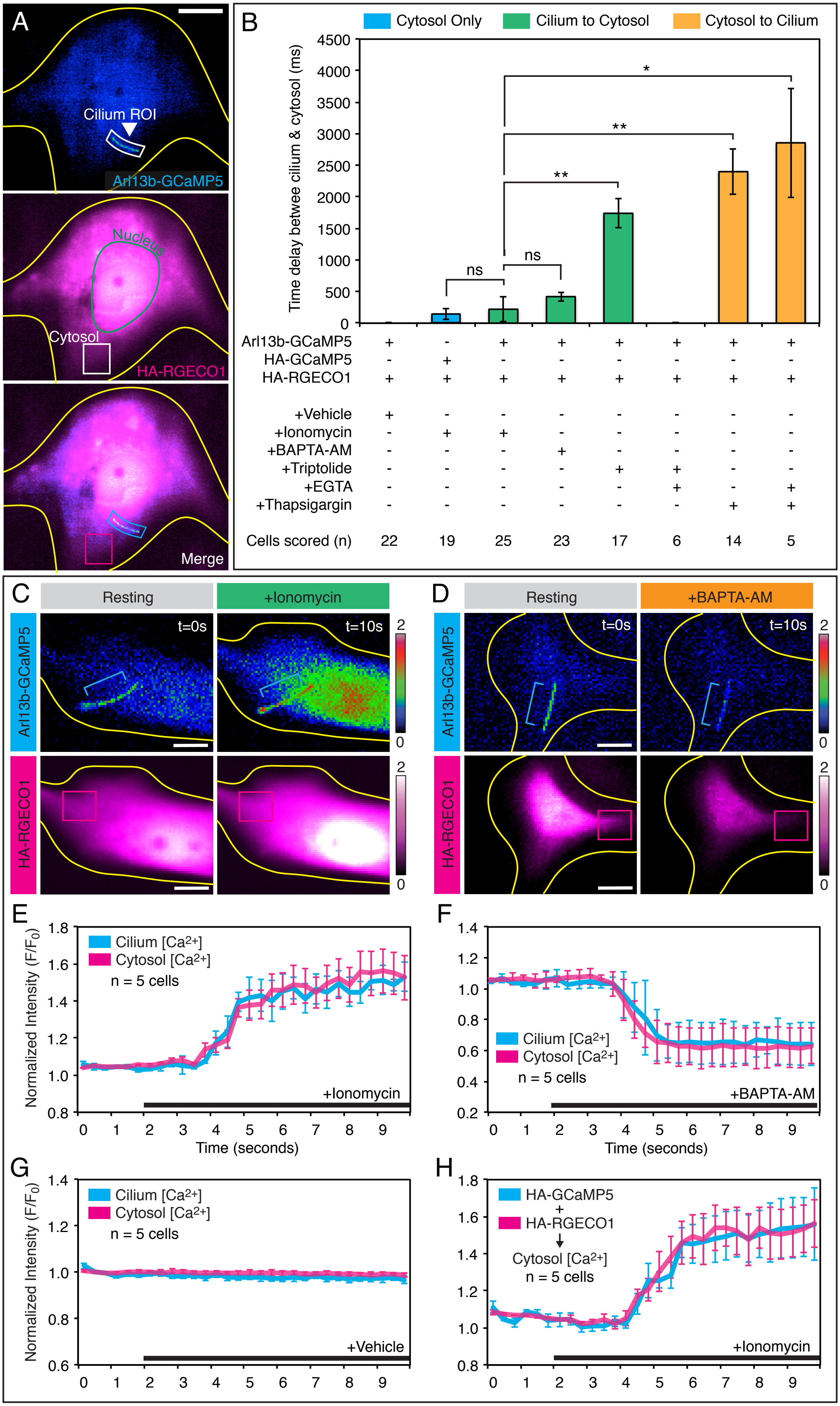

A
Cilium ROI
Arl13b-GCaMP5
Nucleus
Cytosol
HA-RGECO1
Merge
B
Cytosol Only
Cilium to Cytosol
Cytosol to Cilium
Time delay betwee cilium & cytosol (ms)
4500
4000
3500
3000
2500
2000
1500
1000
500
0
*
**
**
ns
ns
Arl13b-GCaMP5 + - + + + + + +
HA-GCaMP5 - + - - - - - -
HA-RGECO1 + + + + + + + +
+Vehicle + - - - - - - -
+Ionomycin - + + - - - - -
+BAPTA-AM - - - + - - - -
+Triptolide - - - - + + - -
+EGTA - - - - - + - +
+Thapsigargin - - - - - - + +
Cells scored (n) 22 19 25 23 17 6 14 5
C
Resting
+Ionomycin
Arl13b-GCaMP5
HA-RGECO1
t=0s
t=10s
2
0
D
Resting
+BAPTA-AM
Arl13b-GCaMP5
HA-RGECO1
t=0s
t=10s
2
0
E
Normalized Intensity (F/F0)
Cilium [Ca2+]
Cytosol [Ca2+]
n = 5 cells
+Ionomycin
Time (seconds)
F
Cilium [Ca2+]
Cytosol [Ca2+]
n = 5 cells
+BAPTA-AM
Time (seconds)
G
Normalized Intensity (F/F0)
Cilium [Ca2+]
Cytosol [Ca2+]
n = 5 cells
+Vehicle
H
HA-GCaMP5
+
HA-RGECO1
Cytosol [Ca2+]
n = 5 cells
+Ionomycin

Figure S1. Pharmacological manipulation of intraciliary and cytosolic calcium levels (Related to Figure 1). A. A single ciliated LLC-PK1 cell co-transfected with *arl13b-GCaMP5* (cyan) and *HA-RGECO1* (magenta). Merged image shows regions of interest (ROI) indicating the cilium and cytosol. The strong HA-RGECO1 background signal in the nucleus identifies transfected cells. Notably, an area outside of the nucleus is used for the cytosolic recording. Cell body is outlined in yellow. **B.** Mean quantified time delay between the initiation of intraciliary and cytosolic calcium fluxes in response to vehicle, ionomycin, BAPTA-AM, triptolide, triptolide/EGTA, thapsigargin or thapsigargin/EGTA from multiple cell recordings. All data is presented as mean±SEM. Triptolide: **P=0.0012; thapsigargin: **P=0.0008; thapsigargin/EGTA: *P=0.0077. ns: P>0.05. **C-D.** Representative images a single LLC-PK1 cell expressing *arl13b-GCaMP5* in the cilium (cyan bracket) and *HA-RGECO1* in the cytosol (magenta box) at resting state and in response to ionomycin (C) or BAPTA-AM (D) treatment. **E-G.** Graphs depict intensity versus time plot from the mean of five independent cell recordings of intraciliary (cyan) and cytosolic (magenta) calcium fluxes recorded in LLC-PK1 cells in response to ionomycin (E), BAPTA-AM (F), and vehicle-only (G). **H.** Graph depicting an intensity versus time plot from the mean of five independent cell recordings of HA-GCaMP5 (cyan) and HA-RGECO1 (magenta) cytosolic calcium fluxes recorded in LLC-PK1 cells in response to ionomycin as a control for reporter kinetic differences. Duration of compound treatment is identified by the black bar. Data in mean recordings represent mean±SEM. All quantified intensity is shown as F/F_0. Scalebars = 10 μm

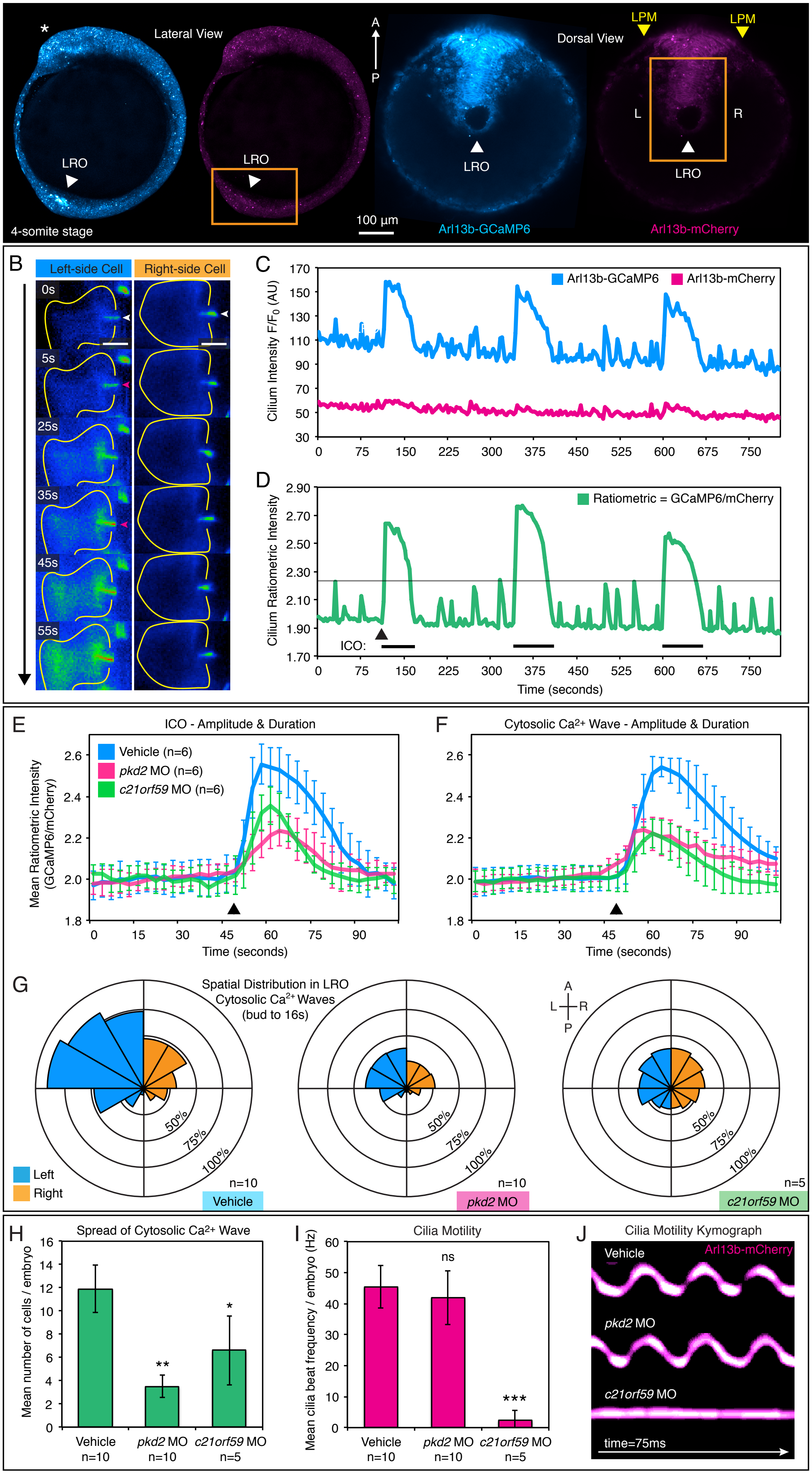

Lateral View
4-somite stage
LRO
A
P
Dorsal View
LPM
L
R
100 µm
Arl13b-GCaMP6
Arl13b-mCherry
B
Left-side Cell
Right-side Cell
0s
5s
25s
35s
45s
55s
C
Arl13b-GCaMP6
Arl13b-mCherry
Cilium Intensity F/F0 (AU)
D
Ratiometric = GCaMP6/mCherry
Cilium Ratiometric Intensity
ICO:
Time (seconds)
E
ICO - Amplitude & Duration
Vehicle (n=6)
pkd2 MO (n=6)
c21orf59 MO (n=6)
Mean Ratiometric Intensity (GCaMP6/mCherry)
Time (seconds)
F
Cytosolic Ca2+ Wave - Amplitude & Duration
Time (seconds)
G
Spatial Distribution in LRO Cytosolic Ca2+ Waves (bud to 16s)
50%
75%
100%
Left
Right
n=10
Vehicle
n=10
pkd2 MO
n=5
c21orf59 MO
H
Spread of Cytosolic Ca2+ Wave
Mean number of cells / embryo
Vehicle n=10
pkd2 MO n=10
c21orf59 MO n=5
I
Cilia Motility
Mean cilia beat frequency / embryo (Hz)
ns
Vehicle n=10
pkd2 MO n=10
c21orf59 MO n=5
J
Cilia Motility Kymograph
Vehicle
Arl13b-mCherry
pkd2 MO
c21orf59 MO
time=75ms

Figure S2. **Ratiometric imaging of ICOs and cytosolic calcium waves in the LRO (Related to Figure 2)**. **A.** Representative fluorescent images of zebrafish embryos co-expressing *arl13b-GCaMP6* and *arl13b-mCherry* at the 4-somite stage (lateral and dorsal views, respectively). The tailbud is outlined in orange, the LRO is highlighted by the white arrowhead, lateral plate mesoderm (LPM) is indicated by yellow arrowhead and the embryo head is noted with an asterisk**.** Scalebar, 100 μm. **B.** Representative montage of ratiometric fluorescent images (Arl13b-GCaMP6/mCherry) of a single wildtype LRO cell from the left (blue) or right-side (orange) of the organ over 55 seconds of total elapsed time. White arrowheads indicate cilia at resting state, while magenta arrowheads indicate onset of ICO. Scalebars, 5 μm. **C.** Two-color fluorescent intensity (F/F_0) over time plot from a single representative LRO cilium (left-side of the organ) co-expressing *arl13b-GCaMP6* and *arl13b-mCherry* at the 1-somite stage. Multiple ICOs are visible in the Arl13b-GCaMP6 (cyan) channel, while the Arl13b-mCherry (magenta) signal is relatively quiescent. **D.** Ratiometric intensity (Arl13b-GCaMP6/mCherry; green) over time plot of the same data in the previous graph. An arrowhead and black bars indicate the start of multiple ICOs. The ratiometric signal was thresholded (indicated by black horizontal line) to facilitate analysis of calcium oscillation dynamics. **E.** Ratiometric intensity (arl13b-GCaMP6/mCherry) plot of a single cilium from a mean of 6 embryos exhibiting ICOs in vehicle (vehicle), *pkd2* MO (magenta) and *c21orf59* MO (green) LROs at the 1-4 somite stage. Each plot was aligned post-acquisition to the first detected ICO (indicated by black arrowhead). **F.** Ratiometric intensity (arl13b-GCaMP6/mCherry) plot of a cell from a mean of 6 embryos exhibiting cytosolic calcium waves in vehicle, *pkd2* MO (cyan) and *c21orf59* MO (magenta) LROs at the 5-9 somite stage. **G.** Rose diagrams depicting the spatial distribution and mean percentage of cells per embryo displaying cytosolic calcium waves in the total LRO of vehicle (vehicle), *pkd2* MO (magenta) and *c21orf59* MO (green) embryos spanning the entire course of LRO development (bud to 16-somite stage). Rings correlate with mean percentage of cells exhibiting calcium waves per embryo from bud

to 16-somite stage (n = 10 embryos at all stages for *pkd2* and vehicle; n = 5 embryos for *c21orf59* and vehicle). Control and experimental samples were acquired in a pair-wise manner and analysis was performed on time-lapse recordings spanning bud to 16-somite stage. Control samples for *c21orf59* morphants are not shown as the distribution and frequency was equivalent to controls for *pkd2* morphants. Left-sided waves, blue. Right-sided waves, orange. **H.** Graph showing the quantified number of cells exhibiting cell-to-cell calcium waves initiated by LRO cilia in vehicle, *pkd2* morphant (n=10 embryos, **P=0.0031) and *c21orf59* morphant (n=5 embryos, *P=0.0179) embryos. All quantified data shown as mean±SEM. **I.** Graph depicting mean LRO ciliary beat frequency (Hz or beats per second) per embryo in vehicle control, *pkd2* knockdown and *c21orf59* knockdown embryos at the 6-8 somite stage. **J.** LRO cilia were labeled by expression of *arl13b-mCherry* and motility was visualized utilizing high-speed fluorescent microscopy. Ciliary waveform kymograph analysis was performed post-acquisition and revealed a sinusoidal pattern for ciliary beat frequency in vehicle and *pkd2* embryos, while *c21orf59* cilia were paralyzed and lacked this pattern. ***P = 0.0001; NS = not significant. A: Anterior, P: Posterior, L: Left, R: Right.

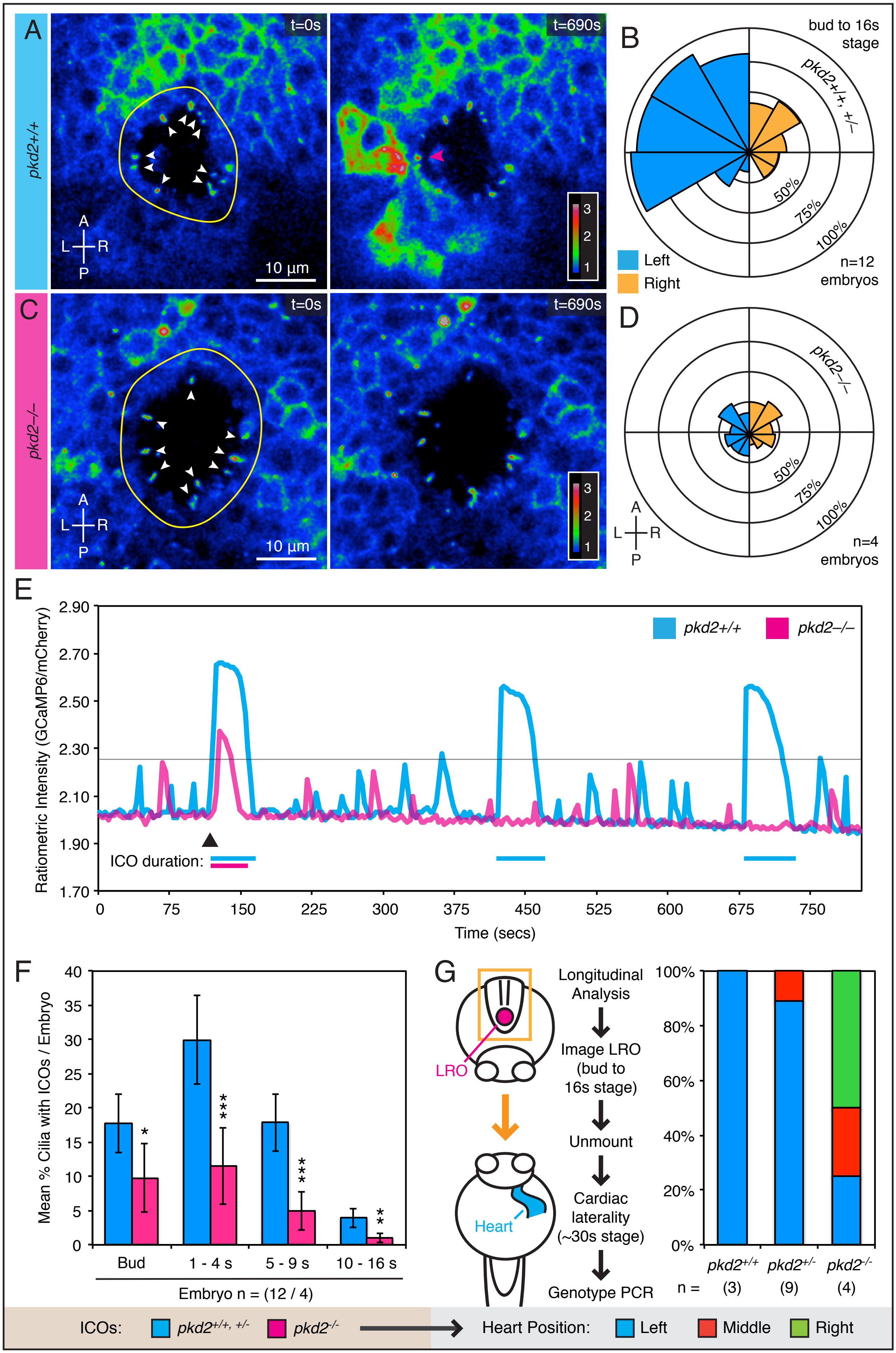

A
pkd2+/+
t=0s
t=690s
A
L R
P
10 µm
3
2
1
B
bud to 16s stage
pkd2+/+, +/–
50%
75%
100%
Left
Right
n=12 embryos
C
pkd2–/–
t=0s
t=690s
10 µm
D
pkd2–/–
n=4 embryos
E
Ratiometric Intensity (GCaMP6/mCherry)
2.90
2.70
2.50
2.30
2.10
1.90
1.70
pkd2+/+
pkd2–/–
ICO duration:
0
75
150
225
300
375
450
525
600
675
750
Time (secs)
F
Mean % Cilia with ICOs / Embryo
40
35
30
25
20
15
10
5
0
Bud
1 - 4 s
5 - 9 s
10 - 16 s
Embryo n = (12 / 4)
G
LRO
Heart
Longitudinal Analysis
Image LRO (bud to 16s stage)
Unmount
Cardiac laterality (~30s stage)
Genotype PCR
100%
80%
60%
40%
20%
0%
pkd2+/+
pkd2+/-
pkd2-/-
n =
(3)
(9)
(4)
ICOs:
pkd2+/+, +/-
pkd2-/-
Heart Position:
Left
Middle
Right

Figure S3. **ICOs are suppressed in *pkd2* mutants (Related to Figure 2). A-D.** Spatial mapping of ICOs in *pkd2* mutants. (A, C) Representative fluorescent live images of LRO from *pkd2*$^{+/+}$ (A), and *pkd2*$^{-/-}$ (C) zebrafish embryos at the 1-somite stage expressing ratiometric GECI (arl13b-GCaMP6/mCherry; ratiometric signal shown and false-colored with rainbow intensometric scale). White arrowheads indicate LRO cilia at resting state, while magenta arrowhead (A) highlights an ICO inducing a cytosolic calcium wave. (B, D) Rose diagrams depicting the spatial distribution and mean percentage of cells per embryo displaying ICOs in the total LRO of *pkd2*$^{+/+,\ +/-}$ (B), and *pkd2*$^{-/-}$ (D) embryos spanning the entire course of LRO development (bud to 16-somite stage). Rings correlate with mean percentage of cells exhibiting calcium oscillations per embryo from bud to 16-somite stage (n = 3 embryos at all stages for *pkd2*$^{+/+}$, n = 9 for *pkd2*$^{+/-}$ and n = 4 for *pkd2*$^{-/-}$). Left-sided calcium, blue. Right-sided calcium, orange. **E.** ICO frequency, duration and amplitude are diminished in *pkd2* mutants. Ratiometric intensity over time plot of a single cilium exhibiting ICOs in *pkd2*$^{+/+}$ (cyan), and *pkd2*$^{-/-}$ (magenta) LROs at the 1-4 somite stage. Each plot was aligned post-acquisition to the first detected ICOs (indicated by black arrowhead) and thresholded (indicated by black horizontal line) to facilitate analysis of calcium oscillation dynamics. A: Anterior, P: Posterior, L: Left, R: Right. Scalebars in A,C: 10 µm. **F.** Temporal mapping of mean percentage of cells exhibiting ICOs per embryo in the entire LRO in *pkd2*$^{+/+,\ +/-}$ (cyan), and *pkd2*$^{-/-}$ (magenta) embryos across the entire course of LRO development (bud to 16-somite). Data is shown as mean±SEM**. G**. Schematic and graph depicting longitudinal analysis of ICOs and cardiac laterality in *pkd2* mutants. Each embryo was imaged for LRO ICOs from bud to 16s stage, and then subsequently analyzed for cardiac looping at ~30s stage. Graph represents percentage of *pkd2*$^{+/+}$, *pkd2*$^{+/-}$ and *pkd2*$^{-/-}$ embryos displaying normal left-sided (blue) or abnormal right-sided (green) or middle (red) positioned hearts.

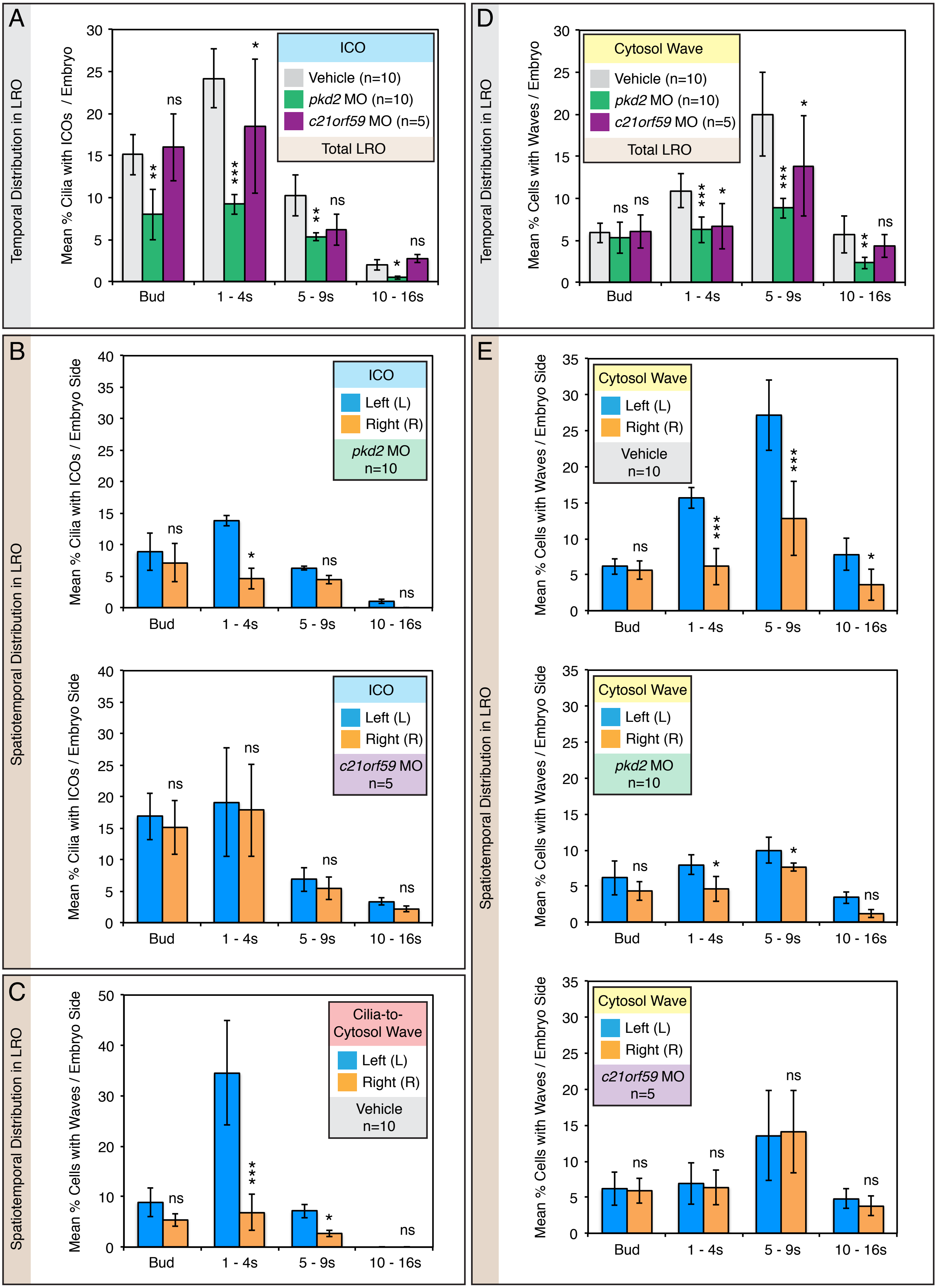
A
Temporal Distribution in LRO
Mean % Cilia with ICOs / Embryo
ICO
Vehicle (n=10)
pkd2 MO (n=10)
c21orf59 MO (n=5)
Total LRO
Bud
1 - 4s
5 - 9s
10 - 16s
D
Temporal Distribution in LRO
Mean % Cells with Waves / Embryo
Cytosol Wave
Vehicle (n=10)
pkd2 MO (n=10)
c21orf59 MO (n=5)
Total LRO
B
Spatiotemporal Distribution in LRO
Mean % Cilia with ICOs / Embryo Side
ICO
Left (L)
Right (R)
pkd2 MO
n=10
c21orf59 MO
n=5
E
Spatiotemporal Distribution in LRO
Mean % Cells with Waves / Embryo Side
Cytosol Wave
Left (L)
Right (R)
Vehicle
n=10
pkd2 MO
n=10
c21orf59 MO
n=5
C
Spatiotemporal Distribution in LRO
Mean % Cells with Waves / Embryo Side
Cilia-to-Cytosol Wave
Left (L)
Right (R)
Vehicle
n=10

Figure S4. ICOs precede cytosolic calcium waves during LRO development (Related to Figure 3). A. Graph depicting the mean percentage of cilia per embryo exhibiting ICOs in the total LRO of vehicle (gray), *c21orf59* morphant (violet), and *pkd2* morphant (green) embryos across the bud to 16-somite stage. **B.** Graphs comparing left-sided (blue) versus right-sided (orange) ICOs at the LRO in *pkd2* MO (green) and *c21orf59* MO (violet) embryos over the entire course of LRO development. **C.** Graph comparing left-sided (blue) versus right-sided (orange) ICOs that are coupled to cytosolic calcium waves at the LRO in vehicle embryos over the entire course of LRO development. **D.** Graph depicting the mean percentage of cells per embryo exhibiting cytosolic calcium waves in the total LRO of vehicle (gray), *c21orf59* morphant (violet), and *pkd2* morphant (green) embryos across the bud to 16-somite stage. **E.** Graphs comparing left-sided (blue) versus right-sided (orange) ICOs at the LRO in vehicle (grey), *pkd2* MO (green) and *c21orf59* MO (violet) embryos over the entire course of LRO development. Control and experimental samples were acquired in a pair-wise manner and analysis was performed on time-lapse recordings spanning bud to 16-somite stage that were segmented by developmental stage. Control samples for *c21orf59* morphants are not shown as the distribution and frequency was equivalent to controls for *pkd2* morphants. All quantified data shown is mean±SEM. NS = not significant. Single asterisk indicates a comparison that results in $P < 0.05$, while double asterisk and triple asterisk indicate $P < 0.005$ and $P < 0.0005$, respectively.

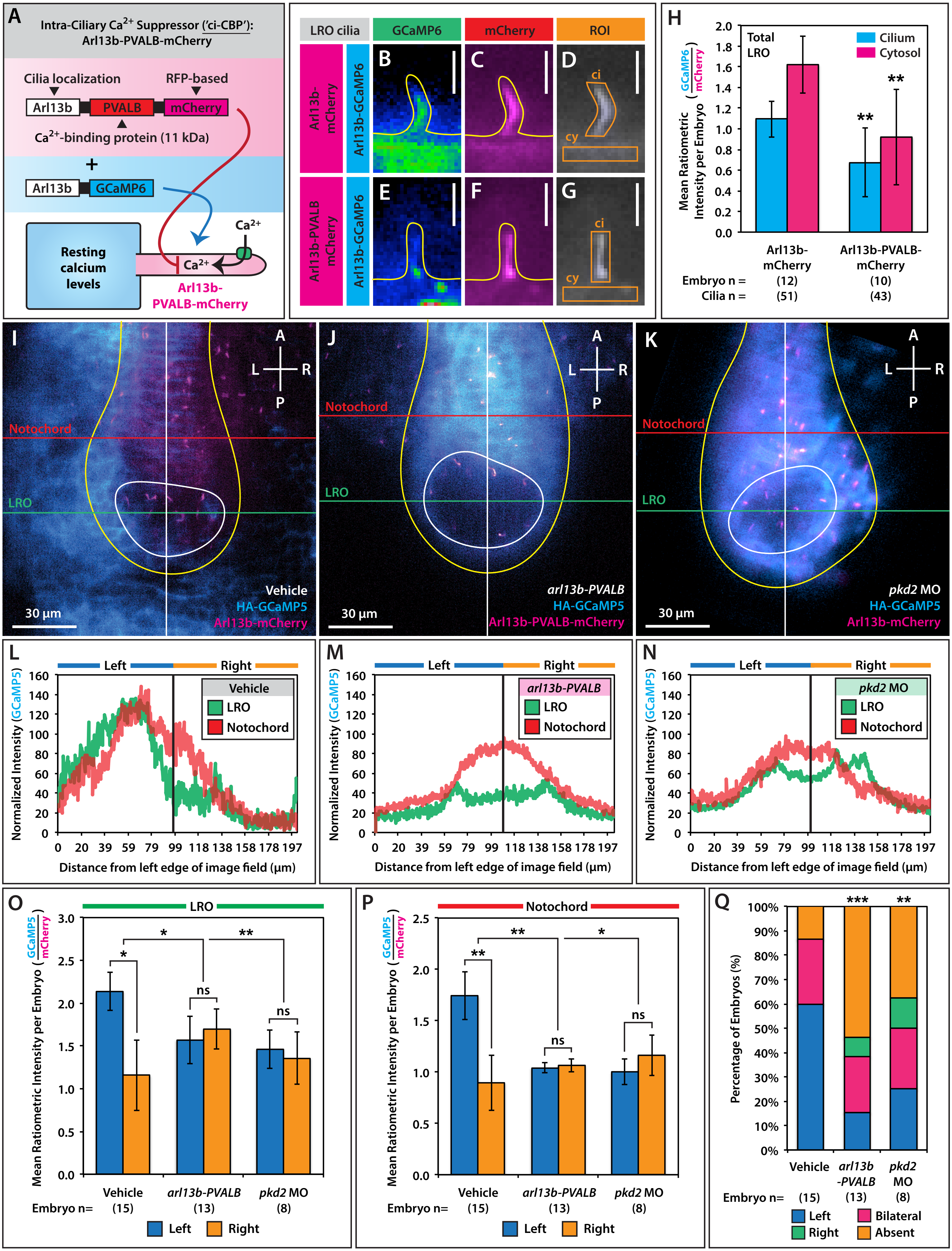

A
Intra-Ciliary Ca2+ Suppressor ('ci-CBP'): Arl13b-PVALB-mCherry
Cilia localization
RFP-based
Arl13b
PVALB
mCherry
Ca2+-binding protein (11 kDa)
+
Arl13b
GCaMP6
Resting calcium levels
Ca2+
Arl13b-PVALB-mCherry
LRO cilia
GCaMP6
mCherry
ROI
Arl13b-mCherry
Arl13b-GCaMP6
Arl13b-PVALB mCherry
B
C
D
E
F
G
ci
cy
H
Total LRO
Cilium
Cytosol
Mean Ratiometric Intensity per Embryo (GCaMP6/mCherry)
Arl13b-mCherry
Arl13b-PVALB-mCherry
Embryo n = (12) (10)
Cilia n = (51) (43)
I
J
K
A
L
R
P
Notochord
LRO
Vehicle
arl13b-PVALB
pkd2 MO
HA-GCaMP5
Arl13b-mCherry
Arl13b-PVALB-mCherry
30 µm
L
M
N
Left
Right
Normalized Intensity (GCaMP5)
Distance from left edge of image field (µm)
O
P
Q
Notochord
Mean Ratiometric Intensity per Embryo (GCaMP5/mCherry)
Vehicle
arl13b-PVALB
pkd2 MO
Embryo n= (15) (13) (8)
Percentage of Embryos (%)
Bilateral
Absent

Figure S5. Suppression of intraciliary calcium by *arl13b-Pvalb* and equimolar expression relative to *HA-Pvalb* (Related to Figure 4). **A**. In vivo strategy for simultaneous suppression and visualization of intraciliary calcium by Arl13b-PVALB-mCherry (cilia-targeted calcium binding protein: 'ci-CBP') and Arl13b-GCaMP6 ('ci-GECI') in LRO cilia. **B.** Disruption of resting LRO intraciliary calcium by Arl13b-PVALB. Representative fluorescent images of immotile LRO cilia from live zebrafish at the 6-somite stage coexpressing *arl13b-GCaMP6* (cyan) and *arl13b-mCherry* (magenta) or *arl13b-Pvalb-mCherry* (magenta). Scalebars = 5 μm. **C.** Rose diagrams depicting the spatial distribution and mean percentage of cells per embryo displaying ciliary-to-cytosolic waves and cytosolic calcium waves in the LRO of *arl13b* (gray) and *arl13b-Pvalb* (magenta) embryos spanning the entire course of LRO development (bud to 16-somite stage; n = 5 for all embryos). Left-sided calcium, blue. Right-sided calcium, orange. **D.** Temporal distribution of ciliary-to-cytosol and cytosolic waves. Graphs depicting mean percentage of cells per embryo exhibiting ciliary-to-cytosol and cytosolic calcium waves in the LRO of *arl13b* and *arl13b-Pvalb* embryos, segmented by developmental stage. Control and experimental samples were acquired in a pair-wise manner and analysis was performed on time-lapse recordings spanning bud to 16-somite stage. **E**. Western blot of whole embryo lysate from embryos microinjected with equimolar concentrations of in vitro transcribed mRNA for *arl13b-mCherry*, *arl13b-Pvalb-mCherry*, *arl13b-Pvalb-CD-mCherry* (contains point mutation in calcium binding domain), and *HA-Pvalb-mCherry*. Blot is probed with antibodies against mCherry and β-tubulin (as loading control). Vehicle embryos were injected with dH_2O. **F-G**. Quantification of Arl13b-PVALB-mCherry and HA-PVALB-mCherry fluorescence in the cytosol of ciliated zebrafish LRO cells. (F) Graph depicting the relative increase of mCherry fluorescence (corrected by cell volume) in the cytosol of embryos expressing *HA-Pvalb-mCherry* relative to *arl13b-Pvalb-mCherry*. Data was quantified from 3D volumetric reconstructions of fluorescent micrographs.

***P<0.0001, n = 22 cells. (G) Maximal intensity projection of Z-stacks through the LRO of embryos co-expressing *mem-EGFP* (not shown) and *arl13b-Pvalb-mCherry* or *HA-Pvalb-mCherry* (magenta) and stained for acetylated-α-tubulin (yellow). Scalebars = 10 μm. **H.** Representative images of whole-mount *in situ* hybridization for *southpaw (spaw)* expression in the lateral plate mesoderm of zebrafish embryos at the 20 somite stage. The embryo is seen from the dorsal side, showing normal left expression (left) or abnormal bilateral (red), right (green) and absent (orange) expression. Graph shows distribution of *spaw* expression domain in response to expression of PVALB and mutant parvalbumin (PVALB-CD) targeted to cilia or cytoplasm. Data shown is pooled from three independent experiments. Statistical comparison was analyzed by one-way ANOVA with Tukey's multiple comparison test. *P < 0.05, while ** P < 0.005, ***P < 0.0005, respectively. NS (not significant): $P \geq 0.05$. Asterisks above brackets denote comparisons between samples indicated by the bracket, while asterisks above a single sample denote comparisons between that sample and the control. n = Total number of embryos analyzed for each experimental condition (in parentheses). Quantified data shown in D and F are mean±SEM.

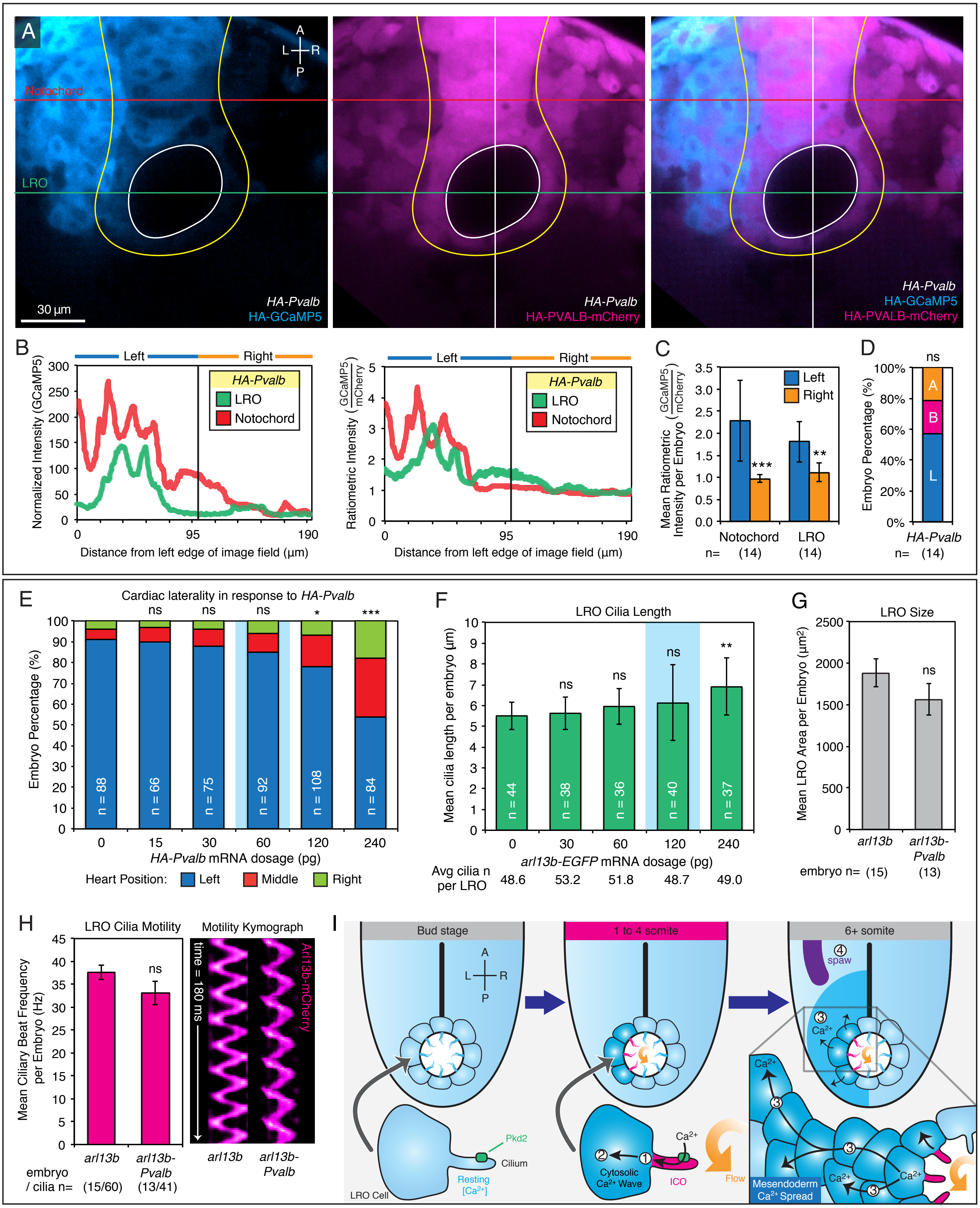

A
A
L
R
P
Notochord
LRO
30 µm
HA-Pvalb
HA-GCaMP5
HA-Pvalb
HA-PVALB-mCherry
HA-Pvalb
HA-GCaMP5
HA-PVALB-mCherry
B
Left
Right
Normalized Intensity (GCaMP5)
HA-Pvalb
LRO
Notochord
Distance from left edge of image field (µm)
Ratiometric Intensity (GCaMP5/mCherry)
Distance from left edge of image field (µm)
C
Mean Ratiometric Intensity per Embryo (GCaMP5/mCherry)
Left
Right
***
**
Notochord
LRO
n= (14) (14)
D
ns
Embryo Percentage (%)
A
B
L
HA-Pvalb
n= (14)
E
Cardiac laterality in response to HA-Pvalb
ns ns ns * ***
Embryo Percentage (%)
n = 88
n = 66
n = 75
n = 92
n = 108
n = 84
HA-Pvalb mRNA dosage (pg)
Heart Position: Left Middle Right
F
LRO Cilia Length
Mean cilia length per embryo (µm)
ns ns ns **
n = 44
n = 38
n = 36
n = 40
n = 37
arl13b-EGFP mRNA dosage (pg)
Avg cilia n per LRO 48.6 53.2 51.8 48.7 49.0
G
LRO Size
Mean LRO Area per Embryo (µm2)
ns
arl13b
arl13b-Pvalb
embryo n= (15) (13)
H
LRO Cilia Motility
Mean Ciliary Beat Frequency per Embryo (Hz)
ns
arl13b
arl13b-Pvalb
embryo / cilia n= (15/60) (13/41)
Motility Kymograph
time = 180 ms
Arl13b-mCherry
arl13b
arl13b-Pvalb
I
Bud stage
1 to 4 somite
6+ somite
A
L
R
P
④ spaw
③ Ca2+
Pkd2
Cilium
Resting [Ca2+]
LRO Cell
② ①
Cytosolic Ca2+ Wave
Ca2+
ICO
Flow
Ca2+
③
Mesendoderm Ca2+ Spread

Figure S6. Intraciliary calcium is upstream of mesendodermal calcium; LRO size and cilia motility are not affected by Arl13b-PVALB (Related to Figure 5). A. Representative fluorescent images of embryo co-expressing *HA-GCaMP5* (cyan) and *HA-Pvalb-mCherry* (magenta) at the LRO during the 6-8 somite stage. Left-sided mesendodermal calcium is undisturbed at a dose of 60 pg of *HA-Pvalb-mCherry* mRNA (merged). **B.** Normalized (GCaMP5) and ratiometric (GCaMP5/mCherry) intensity plot for the images shown in A, showing left-sided mesendodermal calcium at the LRO and surrounding notochord tissue in *HA-Pvalb* embryos. **C.** Quantification of left-sided mesendodermal calcium (measured ratiometrically) across multiple embryos expressing *HA-GCaMP5* and *HA-Pvalb-mCherry* (n = 14). ***P=0.0003, **P=0.0042. **D.** Bargraph depicting the frequency of left-sided (L; blue)), bilateral (B; magenta) or absent (A; orange) mesendodermal calcium in *HA-GCaMP5* / *HA-Pvalb-mCherry* embryos (60 pg; n = 14). ns, P=0.2333 (compared to vehicle in Figure 4E). A: Anterior, P: Posterior, L: Left, R: Right. Scalebars = 30 μm. **E.** Bargraph depicting the dose response of zebrafish cardiac laterality at 26 hpf to *Ha-Pvalb-mCherry* mRNA. Notably, at 60 pg, the maximal concentration utilized in this study, cardiac laterality remained undisturbed (NS = 0.1997). However, at significantly higher doses (120 and 240 pg, respectively), significant LR abnormalities were observed. **F.** Quantification of mean LRO cilia length in response to increasing doses of *arl13b-EGFP* mRNA. Notably, at 120 pg, the maximal concentration utilized in this study, a small but statistically insignificant length increase was detected (NS = 0.0910). At 240 pg, a modest and significant cilia length increase was observed (**P = 0.0012). Length measurements were made from 3D volumetric reconstructions of fluorescent confocal Z-stacks of LRO cilia stained with an antibody against anti-acetylated-α-tubulin. **G.** Quantification of mean LRO area (ns, P=0.1880) in *arl13b* and *arl13b-Pvalb* embryos measured by DIC images. **H.** Cilia beat frequency in the LRO of *arl13b* and *arl13b-Pvalb* expressing embryos**.** Representative kymographs of a single beating cilium from *arl13b* control and *arl13b-Pvalb* LROs*.* LRO cilia were visualized by co-expressing *arl13b-mCherry* as a ciliary marker and recorded live via high-speed fluorescent microscopy at the 6-8 somite stage. Kymographs were

generated post-acquisition to analyze motile ciliary waveform. **I.** Model for intraciliary calcium signaling during LR development. At bud stage, cilia in the LRO are primarily immotile and bilateral symmetry is maintained. At the 1 to 4 somite stage, cilia motility initiates and drives left-biased fluid flow, thereby triggering Pkd2-dependent ICOs along the left-side of the LRO. At the 6+ somite stage, ciliary-to-cytosolic LRO waves spread to neighboring cells to establish leftward-biased stable elevations in calcium around the surrounding mesendoderm, which in turn direct asymmetric gene expression (such as *dand5* and *southpaw*) to influence LR patterning.

Supplemental Experimental Procedures

Animal care ethics. All zebrafish experiments were conducted according to Yale Animal Resources Center (YARC) and Institutional Animal Care and Use Committee (IACUC) guidelines.

Zebrafish husbandry. Zebrafish (*Danio rerio*) care and maintenance were followed according to standard protocols [S1]. Wildtype embryos were collected from TAB-5 and TAB-14 adult crosses. The *pkd2* mutant line, *hi4166*, was previously identified from a retroviral insertion screen [S2]. *pkd2* mutant embryos were collected from crosses between heterozygous adult carriers.

Zebrafish microinjection. All transcripts were microinjected into one-cell stage zebrafish embryos using standard protocols [S1, S3]. Embryos were cultured at 28.5°C.

Morpholinos. Anti-sense morpholino oligonucleotides were ordered from Genetools (Oregon, USA). A standard control oligo (5′-CCTCTTACCTCAGTTACAATTTATA-3′) was injected as a control. A previously characterized oligo, designed against the translational initiation site, was used to knockdown zebrafish *pkd2* (5′-AGGACGAACGCGACTGGAGCTCATC-3′ [S2]) and *c21orf59* (5'-CGCGTGTTATATAAATATGAGACTT-3' [S4]). Morpholinos were diluted in sterile water and injected at a dose of 5 ng for *pkd2* and 16 ng for *c21orf59*.

Cloning of GECIs, PVALB and in vitro mRNA transcription. Construction of plasmids for zebrafish *arl13b* was described previously [S5]. Plasmid DNA for intensometric calcium reporters *GCaMP5G* [S6], *GCaMP6S* [S7]and *RGECO1* [S8] were obtained from Addgene (plasmids #31788, 40753 and 32444), courtesy of L. Looger (HHMI Janelia Farms) and R. Campbell (University of Alberta). Plasmid DNA for calcium binding proteins *Pvalb* [S9] and *Pvalb-CD* [S10] were received as a kind gift from A.

Bennett (Yale University School of Medicine). Plasmid DNA for membrane-localized *RFP* was received as a kind gift from C. Chien (University of Utah). All genes of interest were PCR amplified using Phusion polymerase (Qiagen) and recombined into pCS2-based or pCDNA3-based Gateway expression plasmids, courtesy of N. Lawson (University of Massachusetts, Worcester) and C. Chien (University of Utah) for *in vitro* transcription. All reporters, fluorophores and calcium binding proteins were fused to the C-terminus of zebrafish *arl13b* or a HA-epitope tag utilizing standard Gateway recombination techniques. The mMESSAGE mMACHINE SP6 kit (Ambion) was used to synthesize capped mRNA. RNA was stored at -80°C. On the day of microinjection, RNA was diluted, heated at 65°C for 5 minutes to minimize secondary structures and snap cooled on ice.

Cell culture, transfection and live imaging. LLC-PK1 porcine renal cells were received as a kind gift from S. Somlo and S. Fedeles (Yale University School of Medicine). LLC-PK1 cells were cultured in DMEM:F12 growth medium (Invitrogen) containing 5% fetal bovine serum (Invitrogen) and incubated at 37°C with 5% CO_2. Cells were grown to 70% confluency onto circular coverglass fabricated for a Zeiss cell perfusion chamber and transfected utilizing Lipofectamine 2000 (Invitrogen) according to the manufacturer's protocol in serum-free OptiMEM medium (Invitrogen). After 8 hours, transfection media was replaced with DMEM:F12 media and the cells were serum-starved for 48 hours to induce cilia formation. On the day of imaging, media was replaced with fresh DMEM:F12 media lacking phenol red and serum. The coverglass containing the transfected cells were mounted into a perfusion chamber (Zeiss). Alternatively, cells were grown to 70% confluency, transfected and imaged in 8-well coverglass-bottom slide chambers (Nunc). During live imaging, cells were mounted and incubated on an inverted stage fitted with an environmental chamber (Zeiss) that maintained conditions at 37°C with 5% CO_2 throughout the experiment. Live imaging was performed on an inverted 710 DUO/NLO (Zeiss) confocal microscope with a 63x C-Apochromat water objective, a 7-LIVE scanner with multiple high-speed CCD line detectors, a 488 nm laser, and a 561 nm laser.

Calcium imaging and pharmacological manipulations in cells. LLC-PK1 cells were co-transfected with *arl13b-GCaMP5* and *HA-RGECO1* and simultaneous two-color rapid acquisition was performed. For kinetic comparisons of GCaMP5 and RGECO1, both were N-terminally HA-tagged and transfected into the cell body. For pharmacological manipulations, media containing the vehicle (0.5% DMSO), 1 μM ionomycin (Sigma), 10 μM BAPTA-AM (Invitrogen), 0.1 μM triptolide (Calbiochem) or 5 μM thapsigargin (Sigma) was added to the perfusion chamber via a standard gravity input and output setup during continuous imaging. For vehicle, ionomycin and triptolide studies, the concentration of calcium in the media was adjusted to 1 mM. For calcium chelation studies, 10 mM EGTA (JT Baker) was used in conjunction with calcium-free media to lower extracellular calcium to a level approaching 0. All cellular imaging was performed at an acquisition rate of 2 Hz at 512 x 512 pixel resolution with no binning. To calculate F_0, cells at resting state were recorded for 15 seconds and mean intensity was calculated for each channel. F/F_0 values were normalized to the 15 second resting state mean as baseline. For analysis of cytosolic calcium, we selected cytosolic ROIs based on the following criteria: (1) outside the nucleus and peri-nuclear region, (2) in the cytosol approximately 5 to 10 um in distance from the base of the cilium, (3) on the same side of the cell in which the cilium is located and (4) similar region size and morphology. For analysis of intraciliary calcium, we selected cilia ROIs based on the following criteria: (1) cilium is located outside the nucleus, (2) entire axoneme is clearly visible and mostly straight, (3) cilium is laying largely parallel rather than perpendicular to the cell body, (4) ciliary morphology is normal and does not contain bulbs, and (5) ciliary length is within the range of 3 to 20 μm. Of note, we kept the ROI window size consistent within and between all experiments. Fluorescence intensity analysis was performed in Zen 2009 (Zeiss) and ImageJ (NIH).

Zebrafish cardiac laterality analysis. Wildtype zebrafish embryos were microinjected at the 1-cell stage and heart jogging was scored at the 30 to 32 somite stage (24 to 26 hours post fertizilation) by

scoring for heart positioning across the left-right axis under a dissection microscope with brightfield illumination (Leica). The following doses of in vitro transcribed mRNA were used for microinjection into 1-cell stage embryos to achieve an equimolar final concentration: *arl13b-mCherry*, 120 pg; *arl13b-Pvalb-mCherry*, 138 pg; *arl13b-Pvalb-CD-mCherry*, 138 pg; *HA-Pvalb-mCherry*, 60 pg; *HA-Pvalb-CD-mCherry*, 60 pg. The vehicle consisted of RNAse free water. Fluorescent images show in Figure 5A of cardiac looping were acquired from live transgenic *Tg(cmlc2:EGFP)* zebrafish embryos utilizing a standard fluorescent dissection scope (Leica) with a SPOT CCD camera (Diagnostic Instruments, Inc).

Generation of chimeric DFC embryos. For chimeric $DFC^{arl13b\text{-}Pvalb}$ embryos, *arl13b-Pvalb-mCherry* mRNA (173 pg) was microinjected into the yolk of embryos at the 1000 cell stage (3 hours post fertilization) using previously described methods [S11]. Positive DFC chimeras were sorted for the presence of mCherry fluorescence in the DFCs at 50 to 70% epiboly stage using a fluorescent dissection microscope (Leica). To address the specificity of PVALB in the cilium, $DFC^{HA\text{-}Pvalb}$ embryos were generated by microinjection of *HA-Pvalb-mCherry* mRNA (75 ng) into the yolk of 1000 cell stage embryos. As a control for the expression of *arl13b*, DFC^{arl13b} embryos were generated by injection of *arl13b-mCherry* mRNA (150 pg) into the yolk of embryos at the 1000 cell stage. To control for the presence of *arl13b-Pvalb* and *HA-Pvalb* in the yolk-alone, $yolk^{arl13b\text{-}Pvalb}$ and $yolk^{HA\text{-}Pvalb}$ embryos were generated by microinjection of *arl13b-Pvalb-mCherry* (173 ng) and *HA-Pvalb-mCherry* (75 ng) mRNA into the yolk of embryos at the sphere stage (4 hours post fertilization) [S11]. Chimeric yolk embryos were selected by fluorescent microscopy for mCherry-positive signal in the yolk only at 50 to 70% epiboly stage [S11].

In situ hybridization. Whole mount *in situ* hybridization was performed as described previously, with minor modifications [S12, 13]. Briefly, zebrafish embryos were fixed in diluted formalin (1:2.7 in PBT)

at room temperature for an hour or at 4°C overnight. Digoxigenin-UTP-labeled RNAs synthesized in vitro were used as probes. Alkaline phosphatase-coupled anti-digoxigenin (Roche) was used to localize hybridized probes. BM Purple AP substrate (Roche) was used as the chromogenic substrate to produce purple/blue precipitates. *spaw* riboprobe was described previously [S14] and scoring was performed at the 18 to 20-somite stage. *dand5 (charon)* riboprobe was described previously and scoring was performed at the 8 to 10-somite stage, when asymmetric expression is robust [S15]. Stained embryos were mounted in glycerol and images were taken on a standard dissection microscope (Leica) with a SPOT CCD camera (Diagnostic Instruments, Inc.).

In vivo imaging of intraciliary calcium oscillations in zebrafish LRO. For visualization of intraciliary calcium oscillations and cytosolic calcium waves during LRO development, control embryos were coinjected with sterile water and an equimolar concentration of *arl13b-GCaMP6* and *arl13b-mCherry* mRNA (78 and 60 pg, respectively) at the 1-cell stage and cultured until the 90% epiboly stage (9 hpf). For *pkd2* studies, embryos were coinjected with 5 ng of *pkd2* morpholino, and the previously indicated doses of mRNA. For *c21orf59* studies, embryos were coinjected with 16 ng of *c21orf59* morpholino and the previously indicated doses of mRNA. For *arl13b-Pvalb* studies, embryos were coinjected with sterile water, 78 pg of *arl13b-GCaMP6* mRNA and 138 pg of *arl13b-Pvalb-mCherry* mRNA. During late epiboly stages, embryos were sorted under a standard fluorescent dissection microscope (Leica) and selected for imaging based on the following criteria: (1) normal gross morphology, (2) global positive expression of reporters throughout the entire embryo based on the mCherry signal (does not exhibit mosaicism), (3) not significantly developmentally delayed (± 1 hour) relative to control embryos, and (4) yolk-sac is intact and not damaged. Embryos were dechorionated using 5 mg/mL pronase (Roche) for 5 minutes and then thoroughly washed 5 times with embryo medium. Embryos were submerged in 1% low melt agarose and mounted into 8-well coverglass-bottom slides (Nunc), with the tailbud mounted down towards the coverglass. Long-term

time-lapse imaging of the LRO dorsally commenced at bud stage (10 hpf), when cilia begin to form in the zebrafish LRO, through 16-somite stage (~16 hpf), when the LRO begins to dissipate. For *pkd2* and *c21orf59* studies, control and experimental samples were imaged in a paired manner. To minimize potential bias, we matched the stages of the embryos and alternated wildtype and morphant embryos in a pairwise fashion during acquisition. To accurately stage embryos under acquisition, sibling embryos were stage-matched to the embryo of interest, mounted in a similar fashion, placed near the microscope stage and periodically scored for somite progression under a standard dissection microscope. Imaging was performed on an inverted 710 DUO/NLO (Zeiss) confocal microscope with a 63x C-Apochromat water objective, a 7-LIVE scanner with multiple high-speed CCD line detectors, a 488 nm laser and a 561 nm laser. The microscope stage was fitted with a heated environmental chamber (Zeiss) that maintained conditions at 28.5°C, the optimal temperature for zebrafish embryonic development. Simultaneous two-channel recordings were captured at an acquisition rate of 0.2 Hz at 512 x 512 pixel resolution with no binning. The acquisition rate was chosen based the duration (~40 sec) and slow frequency (2 to 4 minutes) of the intraciliary calcium oscillations at the 1-4 somite stage, but at the same time balancing this with a need to limit phototoxicity during prolonged time-lapse imaging sessions. Of note, recordings of intraciliary calcium oscillations obtained at 1 Hz revealed no significant differences in frequency, duration, amplitude and total number relative to 0.2 Hz; however, photobleaching became a limiting factor for long-term imaging. Optical sections (5 µm thick) were taken at a plane of focus within the zebrafish LRO that best represented the equator of the organ and that captured the maximal number of cilia along the LR axis. Time-lapse recordings and analysis spanned the entire course of LRO development, from bud to 16-somite stage. As this window spans approximately 6 hours, movies were broken into smaller segments spanning from 30 minutes to 2.5 hours for data management purposes.

Spatiotemporal analysis was performed in Zen 2009 (Zeiss) and ImageJ. Due to length and file size restrictions, an 800 second clip that best represented calcium activity from the 1-4 somite stage was

trimmed from longer duration recordings and assembled into movies for presentation purposes only using Final Cut Pro (Apple). All quantified spatiotemporal analysis of intraciliary calcium oscillations (Figures 2-4, S2-5) was based on complete full-length recordings of the entire course of LRO development (bud to 16-somite stage) from individual embryos or the mean of numerous embryos. Notably, our analysis indicated that an 800 second window was of sufficient length to demonstrate multiple calcium oscillations which are highly dynamic and persist for ~40 sec in duration. Fluorescent data was ratiometrically analyzed by dividing the GCaMP6 signal with mCherry during post-acquisition and subsequently false-colored utilizing an intensometric look-up table ("Rainbow RGB") in ImageJ (NIH). To facilitate classification of calcium dynamics in the LRO, we defined a ratiometric threshold value of >2.25 for positive calcium oscillations and waves based on the following observations: (1) wildtype intraciliary oscillations, which were leftward-biased at the LRO, consistently displayed a peak amplitude value that ranged from approximately 2.3 to 3, a duration of at least 30 seconds, a frequency of at least 2 minutes and a high signal-to-noise ratio in the GCaMP channel; (2) smaller fluctuations in intraciliary calcium were commonly detected that exhibited a amplitude of approximately 2.1 to 2.2 and a brief duration (~5 secs) yet were irregular in frequency and distribution and exhibited a low signal-to-noise ratio in the GCaMP channel. Cytosolic calcium activity was defined utilizing similar criteria.

Longitudinal analysis of intraciliary calcium oscillations and cardiac laterality in *pkd2* mutant zebrafish. Embryos were collected from crosses between adult heterozygous *pkd2* carriers (line *hi4166*) [S2]. Each embryo was coinjected with sterile water and an equimolar concentration of *arl13b-GCaMP6* and *arl13b-mCherry* mRNA (78 and 60 pg, respectively) at the 1-cell stage and cultured until the 90% epiboly stage (9 hpf). *pkd2* embryos were dechorionated with pronase and mounted in 1% low melt agar on coverglass using silicone spacers to form wells [S16]. One embryo was mounted per well and each well was numbered and labeled in order to directly correlate LRO

calcium activity with downstream organ situs. Imaging for intraciliary calcium oscillations was followed as described above from bud to 16 somite stage. Movies were also labeled according to the corresponding embryo and well number. Immediately upon completion of imaging, silicone spacers were removed, each embryo was excised from the agar using watchmaker forceps and a 1.8 mm phaco angled slit knife (Surgistar, Inc.) and placed into individual labeled wells of a 24-well plate. At 30 somite stage (~24 hpf) and 56 hpf, cardiac looping and body axis curvature phenotypes were scored. Notably, *pkd2-/-* zebrafish mutants display randomized cardiac looping and ventral body curvature, while heterozygotes and wildtype embryos do not [S2]. Embryos were then lysed in a solution containing 50 ng/ul proteinase K at 55° for 4 hours. Proteinase K was inactivated by incubating at 95° for 1 hour and the subsequent solution was then used in a multiplex PCR reaction with *hi4166* genotyping primers against the retroviral insertion to identify homozygous mutants from heterozygous mutants and homozygous wildtype embryos. In this manner, calcium activity at the LRO was directly correlated with the development of LR asymmetry in *pkd2* mutant and wildtype embryos. Of note, the genotype of the embryo is unknown during calcium imaging due to the Mendelian nature of the heterozygous cross, thus facilitating a semi-blindfolded analysis of mutants, heterozygotes and wildtype embryos.

Spatial analysis of motile and immotile cilia populations in the zebrafish LRO. Spatial analysis and additive mapping of LRO cilia was quantified as previously described with minor modifications [S17]. Briefly, live high-speed acquisitions of zebrafish LROs from bud to 16-somite stage expressing *arl13b-mCherry* were slowed down, played back, and scored for motile and immotile cilia in ImageJ (NIH) onto two overlaying images: one for motile and the second for immotile cilia. Each overlaying image was converted into a binary image and then converted a second time into a false-colored 8-bit image. Both images were then merged to generate a two-color map of cilia distribution for an individual LRO. For each experimental condition, numerous two-color maps were assembled into an

image series and processed into a single composite image using the projection function in ImageJ. The tailbud morphology surrounding each LRO was traced from corresponding DIC images in ImageJ as a third overlaying image.

Suppression of intraciliary calcium in zebrafish LRO. To characterize the inhibition of intraciliary calcium by Arl13b-PVALB in the zebrafish LRO, experimental embryos were co-injected with 138 pg of *arl13b-Pvalb-mCherry* mRNA and 78 pg of *arl13b-GCaMP6* mRNA. Control embryos were co-injected with 120 pg of *arl13b-mCherry* mRNA and 78 pg of *arl13b-GCaMP6* mRNA. Embryos were mounted as described above in low melt agarose. Calcium imaging and analysis of data was performed as described above. To address equimolar expression of Arl13b-PVALB and HA-PVALB, embryos were injected with 80 pg of *membrane-EGFP* and an equimolar concentration of either *arl13b-Pvalb-mCherry* (138 pg) or *HA-Pvalb-mCherry* (60 pg). Embryos were then fixed in 4% paraformaldehyde overnight at 4°C, washed in PBS-T, blocked in 10% goat serum and incubated with an antibody against acetylated-α-tubulin (Sigma; mouse; 1:5000) for 2 hours at RT. An Alexa647-conjugated secondary antibody (Invitrogen) was utilized to visualize tubulin, while fusion proteins were utilized to visualize PVALB and the cell membrane. Embryos were pre-incubated in Prolong Gold (Invitrogen), mounted in low-melt agarose and Z-stacks through the entire LRO were recorded. The relative amount of total mCherry fluorescence in the cytosol of LRO cells was quantified and normalized to cell volume at the 6-8 somite stage via 3D volumetric reconstructions of LRO cells utilizing Volocity (Perkin Elmers). ROIs were drawn encompassing individual LRO cells by tracing membrane-GFP. All imaging was performed on an inverted 710 DUO/NLO (Zeiss) confocal microscope with a 63x C-Apochromat water objective, a 7-LIVE module with multiple high-speed CCD line detectors a 488 nm laser, a 561 nm laser, and a 633 nm laser.

Mesendodermal calcium imaging and cilia motility analysis in zebrafish LRO. For fine Z-section analysis of asymmetric mesendodermal calcium signaling at the LRO, zebrafish embryos were coinjected with 50 pg membrane-localized *RFP* and 80 pg *HA-GCaMP6* mRNA. To test for the effects of suppressing intraciliary calcium on cytosolic signaling at the LRO, zebrafish embryos were coinjected with 138 pg *arl13b-Pvalb-mCherry* and 80 pg *HA-GCaMP5* mRNA. As a control, zebrafish embryos were coinjected with 120 pg of *arl13b-mCherry* and 80 pg of *HA-GCaMP5* mRNA. For *pkd2* studies, wildtype embryos were coinjected with 5 ng of *pkd2* morpholino, 120 pg of *arl13b-mCherry* mRNA, and 80 pg of *HA-GCaMP5* mRNA. For *HA-Pvalb* studies, wildtype embryos were coinjected with 60 pg of *HA-Pvalb-mCherry* and 80 pg of *HA-GCaMP5* mRNA. Live imaging of the LRO was performed as previously described [S16]. Briefly, embryos were microinjected at the 1-cell stage, then cultured at 28.5°C until the 2-4 somite stage. After manual dechorionation, embryos were mounted in 1.5% low melt agarose on coverglass as previously described. The agar block was then covered with a drop of standard embryo medium and the embryos were cultured until the 6-8 somite stage, when cilia-driven fluid flow in the LRO is well established. Live imaging was performed on an inverted 710 DUO/NLO (Zeiss) confocal microscope with a 63x water C-Apochromat objective, a 7-LIVE scanner with multiple high-speed CCD line detectors, a 488 nm laser and a 561 nm laser. Simultaneous two-channel recordings were captured at an acquisition rate of 120 Hz at 512 x 512 pixel resolution with no binning. Recordings were taken at multiple planes of focus within the zebrafish LRO and analyzed in Zen 2009 (Zeiss) and ImageJ (NIH). Movies were assembled using Final Cut Pro (Apple). To examine for the effects of suppressing intraciliary calcium or *c21orf59* knockdown on cilia motility at the LRO, cilia labeled in the Arl13b-mCherry or Arl13b-PVALB-mCherry channel were analyzed individually by kymographic analysis in ImageJ (NIH) and the mean beat frequency per embryo was calculated [S16]. LRO area was quantified in ImageJ using DIC images by ROIs corresponding to the luminal border of the organ.

Arl13b cilia length measurements in the LRO. For LRO cilia length measurements, embryos were injected with sterile water or the indicated doses of *arl13b-EGFP* mRNA (spanning 30 – 240 pg) at the 1-cell stage. Embryos were dechorionated utilizing watchmakers forceps and collected at the 6-8 somite stage. Embryos were then fixed in 4% paraformaldehyde overnight at 4°C, washed in PBS-T, blocked in 10% goat serum and incubated with an antibodies against acetylated-α-tubulin (Sigma; mouse; 1:5000) and atypical PKC (Santa Cruz, rabbit, 1:100) for 2 hours at RT. Alexa-conjugated secondary antibodies (Invitrogen, 1:500) were utilized to visualize tubulin, while fusions proteins were utilized to visualize Arl13b-EGFP. Embryos were pre-incubated in Prolong Gold (Invitrogen) and mounted in low-melt agarose and Z-stacks through the entire LRO were recorded. Cilia length was measured and quantified from 3D volumetric reconstructions of the Z-stacks using the skeletal length measurement parameter in Volocity (PerkinElmer), as previously detailed [S16, 18]. Renderings of cilia were generated from the volumetric reconstructions using the 3D opacity viewing mode in Volocity. All imaging was performed on an inverted 710 DUO/NLO (Zeiss) confocal microscope with a 63x C-Apochromat water objective, a 488 nm laser, a 561 nm laser, and a 633 nm laser.

Preparation of embryo lysates and western blots. Embryo lysates were prepared with 20-hpf embryos as previously described with minor modifications [S19, 20]. Prepared samples were loaded onto 4–20% Tris–HCl mini gradient gels (BioRad), then transferred to nitrocellulose (BioRad). Immunoblots were carried out using Tris-buffered saline (TBS), pH 7.4, 5% milk, 0.1% Tween 20 for primary and secondary antibodies. After a final wash in TBS, 0.5% Triton X-100, blots were incubated in Western Lightning Chemiluminescence Plus (PerkinElmer LAS, Inc.) and exposed to film. The following primary antibodies were used: rabbit anti-DsRed/mCherry at 1:1000 (Clontech; #632496) and mouse anti-β-tubulin at 1:1000 (Sigma; #T5201). All horseradish peroxidase–conjugated secondary antibodies (Jackson ImmunoResearch Laboratories) were used at 1:5000. All horseradish

peroxidase–conjugated secondary antibodies (Jackson ImmunoResearch Laboratories) were used at 1:5000.

Statistics

In all datasets and figures, the criteria for statistical significance was defined as $P < 0.05$. Data that did not meet these criteria ($P \geq 0.05$) was denoted as not statistically significant ("ns"). Single asterisk indicates a comparison that results in $P < 0.05$, while double asterisk and triple asterisk indicate $P < 0.005$ and $P < 0.0005$, respectively. Asterisks above brackets denote comparisons between samples indicated by the bracket, while asterisks above a single sample denote comparisons between that sample and the control. For spatiotemporal analysis of intraciliary calcium oscillations, ciliary-to-cytosolic waves and cytosolic waves (Figures 3B and S4B,C,E), chi-squared test was utilized to compare observed versus expected frequencies using SPSS (IBM Corporation). The zebrafish LRO, which is mostly spherical, was bisected into two hemispheres: left-side and right-side. We then compared the mean percentage of cells per embryo exhibiting calcium oscillations/waves waves within the left versus right-side to an expected frequency assuming baseline bilateral symmetry (50% left = 50% right). For other experiments, *P*-values were derived from one-way ANOVA analysis and an appropriate post-hoc multi-comparison test using SPSS (IBM Corporation) in experiments with more than 2 means. Dunnett's post-hoc test was performed in Figures 3B, 3C, 4D, 4E, S1B, S2H, S2I, S3F, S4A-E, S5D, S6C-F. Tukey's post-hoc test was performed in Figures 5A, 5B, 6C, S5H. Student's t-test (two-tailed) was performed using SPSS in the following experiments comparing 2 means: Figures S5F, S6G, S6H. All quantified zebrafish data represents mean ± standard error of the mean (SEM) per embryo. All rose diagrams were generated in the statistical program 'R' (www.r-project.org) using the 'ggplot2' graphing package (www.ggplot2.org).

Supplemental References

S1. Westerfield, M. (2000). The zebrafish book. A guide for the laboratory use of zebrafish (Danio rerio). 4th Edition, (Eugene, OR 97403: University of Oregon).

S2. Sun, Z., Amsterdam, A., Pazour, G.J., Cole, D.G., Miller, M.S., and Hopkins, N. (2004). A genetic screen in zebrafish identifies cilia genes as a principal cause of cystic kidney. Development *131*, 4085-4093.

S3. Yuan, S., and Sun, Z. (2009). Microinjection of mRNA and morpholino antisense oligonucleotides in zebrafish embryos. J Vis Exp.

S4. Austin-Tse, C., Halbritter, J., Zariwala, M.A., Gilberti, R.M., Gee, H.Y., Hellman, N., Pathak, N., Liu, Y., Panizzi, J.R., Patel-King, R.S., et al. (2013). Zebrafish Ciliopathy Screen Plus Human Mutational Analysis Identifies C21orf59 and CCDC65 Defects as Causing Primary Ciliary Dyskinesia. American journal of human genetics *93*, 672-686.

S5. Duldulao, N., Lee, S., and Sun, Z. (2009). Cilia localization is essential for in vivo functions of the Joubert syndrome protein Arl13b/Scorpion. Development *136*, 4033-4042.

S6. Akerboom, J., Chen, T.W., Wardill, T.J., Tian, L., Marvin, J.S., Mutlu, S., Calderon, N.C., Esposti, F., Borghuis, B.G., Sun, X.R., et al. (2012). Optimization of a GCaMP calcium indicator for neural activity imaging. The Journal of neuroscience : the official journal of the Society for Neuroscience *32*, 13819-13840.

S7. Chen, T.W., Wardill, T.J., Sun, Y., Pulver, S.R., Renninger, S.L., Baohan, A., Schreiter, E.R., Kerr, R.A., Orger, M.B., Jayaraman, V., et al. (2013). Ultrasensitive fluorescent proteins for imaging neuronal activity. Nature *499*, 295-300.

S8. Zhao, Y., Araki, S., Wu, J., Teramoto, T., Chang, Y.F., Nakano, M., Abdelfattah, A.S., Fujiwara, M., Ishihara, T., Nagai, T., et al. (2011). An expanded palette of genetically encoded Ca(2)(+) indicators. Science *333*, 1888-1891.

S9. Pusl, T., Wu, J.J., Zimmerman, T.L., Zhang, L., Ehrlich, B.E., Berchtold, M.W., Hoek, J.B., Karpen, S.J., Nathanson, M.H., and Bennett, A.M. (2002). Epidermal growth factor-mediated activation of the ETS domain transcription factor Elk-1 requires nuclear calcium. The Journal of biological chemistry *277*, 27517-27527.

S10. Rodrigues, M.A., Gomes, D.A., Leite, M.F., Grant, W., Zhang, L., Lam, W., Cheng, Y.C., Bennett, A.M., and Nathanson, M.H. (2007). Nucleoplasmic calcium is required for cell proliferation. The Journal of biological chemistry *282*, 17061-17068.

S11. Amack, J., and Yost, H. (2004). The T box transcription factor no tail in ciliated cells controls zebrafish left-right asymmetry. Curr Biol *14*, 685-690.

S12. Hauptmann, G., and Gerster, T. (2000). Multicolor whole-mount in situ hybridization. Methods Mol Biol *137*, 139-148.

S13. Thisse, C., and Thisse, B. (2008). High-resolution in situ hybridization to whole-mount zebrafish embryos. Nat Protoc *3*, 59-69.

S14. Long, S., Ahmad, N., and Rebagliati, M. (2003). The zebrafish nodal-related gene southpaw is required for visceral and diencephalic left-right asymmetry. Development *130*, 2303-2316.

S15. Lopes, S.S., Lourenco, R., Pacheco, L., Moreno, N., Kreiling, J., and Saude, L. (2010). Notch signalling regulates left-right asymmetry through ciliary length control. Development *137*, 3625-3632.

S16. Yuan, S., Zhao, L., and Sun, Z. (2013). Dissecting the functional interplay between the TOR pathway and the cilium in zebrafish. Methods in enzymology *525*, 159-189.

S17. Boskovski, M.T., Yuan, S., Pedersen, N.B., Goth, C.K., Makova, S., Clausen, H., Brueckner, M., and Khokha, M.K. (2013). The heterotaxy gene GALNT11 glycosylates Notch to orchestrate cilia type and laterality. Nature *504*, 456-459.

S18. Yuan, S., Li, J., Diener, D.R., Choma, M.A., Rosenbaum, J.L., and Sun, Z. (2012). Target-of-rapamycin complex 1 (Torc1) signaling modulates cilia size and function through protein synthesis regulation. Proc Natl Acad Sci U S A *109*, 2021-2026.

S19. DiBella, L.M., Park, A., and Sun, Z. (2009). Zebrafish Tsc1 reveals functional interactions between the cilium and the TOR pathway. Human molecular genetics *18*, 595-606.

S20. Link, V., Shevchenko, A., and Heisenberg, C.P. (2006). Proteomics of early zebrafish embryos. BMC developmental biology *6*, 1.
